# A positive feedback loop reinforces the allergic immune response in human peanut allergy

**DOI:** 10.1084/jem.20201793

**Published:** 2021-05-04

**Authors:** Xiaoying Zhou, Wong Yu, Shu-Chen Lyu, Claudia Macaubas, Bryan Bunning, Ziyuan He, Elizabeth D. Mellins, Kari C. Nadeau

**Affiliations:** 1 Sean N. Parker Center for Allergy & Asthma Research at Stanford University and Division of Pulmonary, Allergy, and Critical Care Medicine, Stanford, CA; 2 Department of Pediatrics, Program in Immunology, Stanford University, Stanford, CA

## Abstract

Food allergies are a leading cause of anaphylaxis, and cellular mechanisms involving antigen presentation likely play key roles in their pathogenesis. However, little is known about the response of specific antigen-presenting cell (APC) subsets to food allergens in the setting of food allergies. Here, we show that in peanut-allergic humans, peanut allergen drives the differentiation of CD209^+^ monocyte-derived dendritic cells (DCs) and CD23^+^ (FcєRII) myeloid dendritic cells through the action of allergen-specific CD4^+^ T cells. CD209^+^ DCs act reciprocally on the same peanut-specific CD4^+^ T cell population to reinforce Th2 cytokine expression in a positive feedback loop, which may explain the persistence of established food allergy. In support of this novel model, we show clinically that the initiation of oral immunotherapy (OIT) in peanut-allergic patients is associated with a decrease in CD209^+^ DCs, suggesting that breaking the cycle of positive feedback is associated with therapeutic effect.

## Teaser

Peanut-specific CD4^+^ T cells drive the differentiation of CD209^+^ DCs, which then act reciprocally on the same CD4^+^ T cell population to increase Th2 cytokine expression in a positive feedback loop.

## Introduction

Food allergies affect ≤10% of children and adults in different countries around the globe, including the United States (Gupta et al., 2018; Gupta et al., 2019), China (Hu et al., 2010), Australia (Osborne et al., 2011; Tang and Mullins, 2017), Honduras (Gonzales-González et al., 2018), United Arab Emirates (Al-Hammadi et al., 2010), Italy (Asero et al., 2009; Caffarelli et al., 2011), and Poland (Bartuzi et al., 2017; Steinke et al., 2007). Because of the life-threatening potential of anaphylaxis associated with IgE-mediated food allergies, this disease has become a significant public health problem and health cost burden (Gupta et al., 2019).

It has long been understood that IgE-mediated food allergies result from a type 2 helper T cell (Th2) immune response of the adaptive immune system to protein antigens associated with specific foods (Akdis, 2006; Chiang et al., 2018; Sampson et al., 2018; Turcanu et al., 2003; Wambre et al., 2017; Wisniewski et al., 2015; Yu et al., 2016). More recently, our understanding of the factors contributing to food allergy pathogenesis has grown to include epithelial barrier function and signaling (Groschwitz and Hogan, 2009; Wesemann and Nagler, 2016), the host microbiota (Feehley et al., 2019; Wesemann and Nagler, 2016), and the innate immune system (Minnicozzi et al., 2011; Tordesillas et al., 2017).

**Figure 1.**
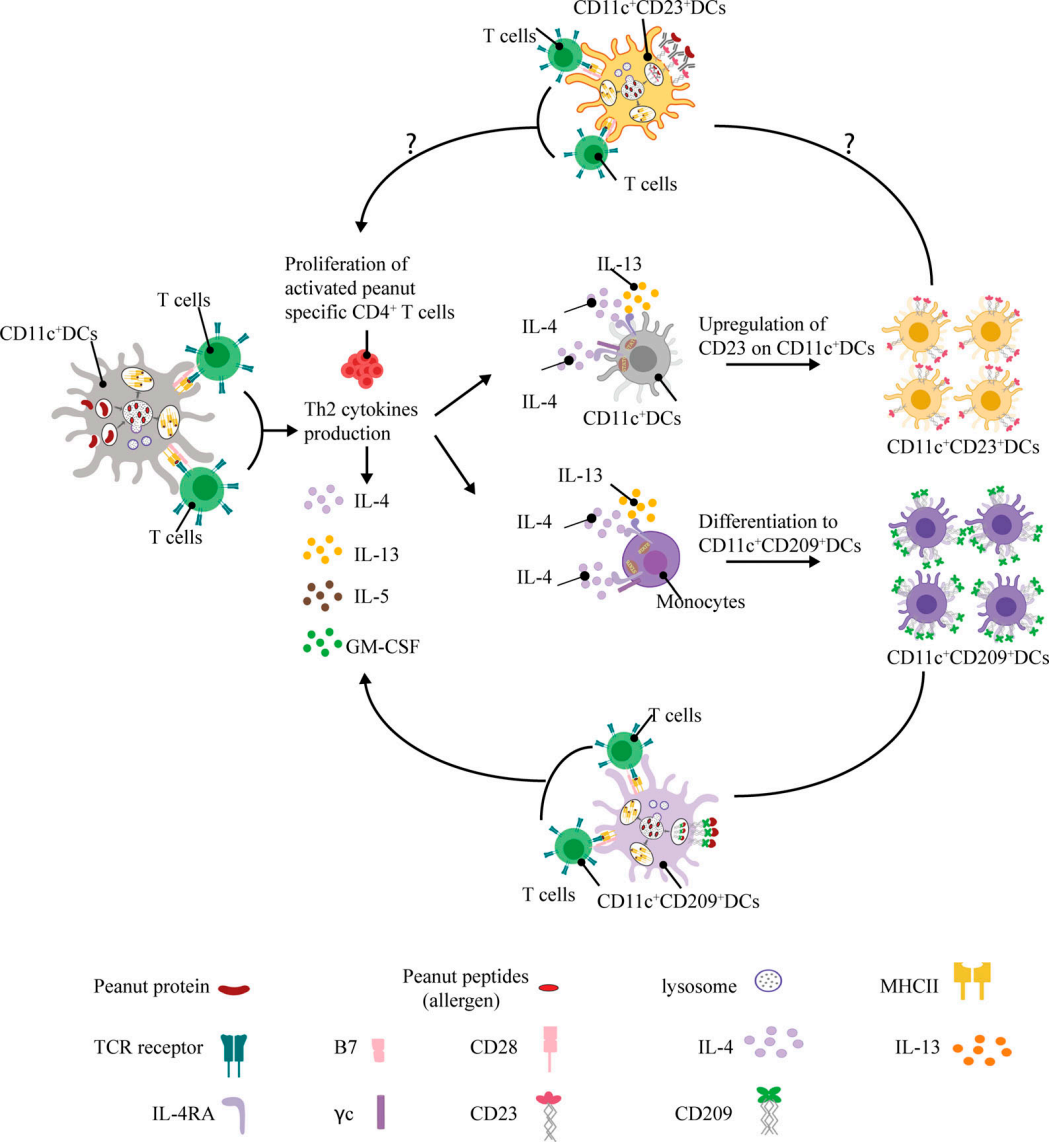
**A positive feedback loop between the adaptive and innate immune systems in food allergy.** Allergen exposure induces antigen-specific CD4^+^ T cells to secrete IL-4 and IL-13 in food-allergic patients. These Th2 cytokines drive the differentiation of monocytes into CD209^+^ MDDCs and the up-regulation CD23 on myeloid CD11c^+^ DCs. This in turn facilitates the uptake and HLA presentation of allergen, reinforcing the adaptive immune response to allergen.

The innate immune system includes cells well known to be involved in food allergy such as mast cells, basophils, group 2 innate lymphoid cells (ILC2s) and dendritic cells (DCs; Minnicozzi et al., 2011; Tordesillas et al., 2017). More recently, evidence for a link between monocytes and the development of food allergy has been reported: cord blood from infants who eventually developed food allergy contained an increased frequency of monocytes which, when stimulated by LPS, secreted greater amounts of the inflammatory cytokines IL-1β, TNF-α, and IL-6 (Zhang et al., 2016). Similarly, 1-yr-old infants with egg allergy had increased numbers of circulating blood monocytes and DCs; LPS stimulation of peripheral blood mononuclear cells (PBMCs) again resulted in a greater secretion of the same inflammatory cytokines (Neeland et al., 2018). LPS, or endotoxin, is a bacterial cell wall product and is commonly found at variable levels in dust from domestic and occupational sources (Yang et al., 2020). Human and mouse studies suggest that exposure to LPS protects against the development of allergic asthma and so has the potential to dampen Th2 immune responses (Hollingsworth et al., 2006; Williams et al., 2005). Although the work by Zhang et al. (2016) and Neeland et al. (2018) examined the response of monocytes to LPS from food-allergic patients, IgE-mediated food allergy results from the immune response to protein antigens associated with specific foods. Thus, LPS-stimulated monocytes are unlikely to be the optimal model for investigating food-allergic patients, in addition to the fact that individuals have different levels of environmental exposure to LPS, and LPS may impair allergic immune responses. To our knowledge, the response of antigen-presenting cells (APCs) to food allergens in established food allergy has not been investigated yet. Understanding the mechanisms underlying the specific response of APCs to food allergens in this setting would facilitate the development of potential targets for therapeutic benefit. Here, we provide experimental evidence for a novel hypothesis (Fig. 1) in which a positive feedback loop between Th2 cells and APCs reinforces the allergic immune response in food allergy.

To gain insight into the role of APCs in the immune response to food allergens, we compared PBMCs in peanut-allergic (PA) and nonallergic (NA) children after stimulation with peanut protein. Using mass cytometry by time of flight (CyTOF) and a Luminex-based 62-multiplex cytokine assay, we observed an increase in monocyte-associated cytokine secretion coupled with a decrease in the frequency of monocytes in PBMCs from PA individuals, but not NA individuals. Using CFSE labeling, antibody blocking, and T cell depletion, we then demonstrated that in PA individuals, allergen exposure stimulates IL-4/IL-13 secretion by CD4^+^ T cells that drives the differentiation of two types of APCs: CD209^+^ monocyte-derived DCs (MDDCs) and myeloid DCs expressing the low-affinity Fc receptor for IgE (FcεRII or CD23). In the case of CD209^+^ MDDCs, the addition of an anti-CD209 blocking monoclonal antibody reduced the frequency of peanut-activated CD4^+^ T cells expressing IL-4 and IL-13 from PA subjects. These results support a model in which allergen exposure in food-allergic individuals results in a positive feedback loop that reinforces the allergic immune response. Consistent with this model, we show that the treatment of PA children with oral immunotherapy (OIT) results in decreased CD209^+^ MDDCs, potentially breaking the positive feedback loop.

## Results

### Food-allergic versus healthy children secrete a distinct cytokine profile after stimulation with allergenic protein

**Figure 2.**
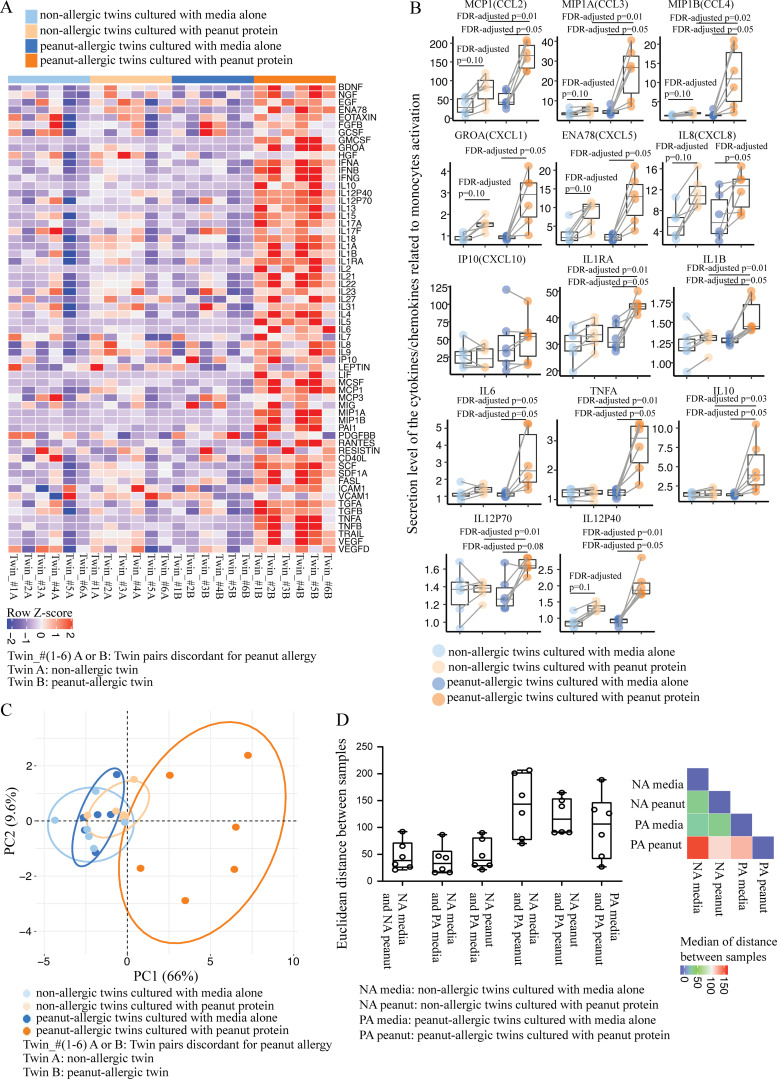
**PBMCs from PA individuals secrete a distinct cytokine profile after stimulation with peanut protein. (A)** Heatmap of secretion levels of 62 cytokines measured by Luminex-based 62-multiplex assay from PBMCs stimulated with or without peanut protein for six pairs of peanut allergy–discordant twins. Each column represents PBMCs from the indicated twin sibling cultured with medium alone or with peanut protein. Red and blue indicate higher and lower expression, respectively. Colored bars at the top of the heatmap indicate peanut allergy status and culture conditions for each experimental group. **(B)** Secreted levels of 14 cytokines related to monocyte activation from PBMCs stimulated with or without peanut protein for six pairs of peanut allergy–discordant twins. Each pair of points connected by a line represents a sample from one sibling twin. Box plots indicate the interquartile range (IQR) and median; whiskers extend to the farthest data point within a maximum of 1.5× IQR. Sets of paired samples were analyzed using the Wilcoxon signed rank test (two sided). Unpaired sample sets were analyzed using the Wilcoxon rank sum test (two sided). P values were adjusted for multiple comparisons using the Benjamini and Hochberg approach to control the FDR. FDR-adjusted P values <0.1 were considered significant. **(C)** Unsupervised PCA of 14 cytokines related to monocyte activation for six pairs of peanut allergy–discordant twins. The percentage variances explained by principal component 1 (PC1) and PC2 are indicated. **(D)** Euclidean distances computed based on 14 cytokines related to monocyte activation for six pairs of peanut allergy–discordant twins. Box plots overlaid with dot plots represent the Euclidean distances calculated pairwise either between samples from same person cultured under different conditions or between samples from twin siblings, depending on the experimental group (six pairs for each group; left); heatmap represents the median values of the distances between each group (right).

**Figure S1.**
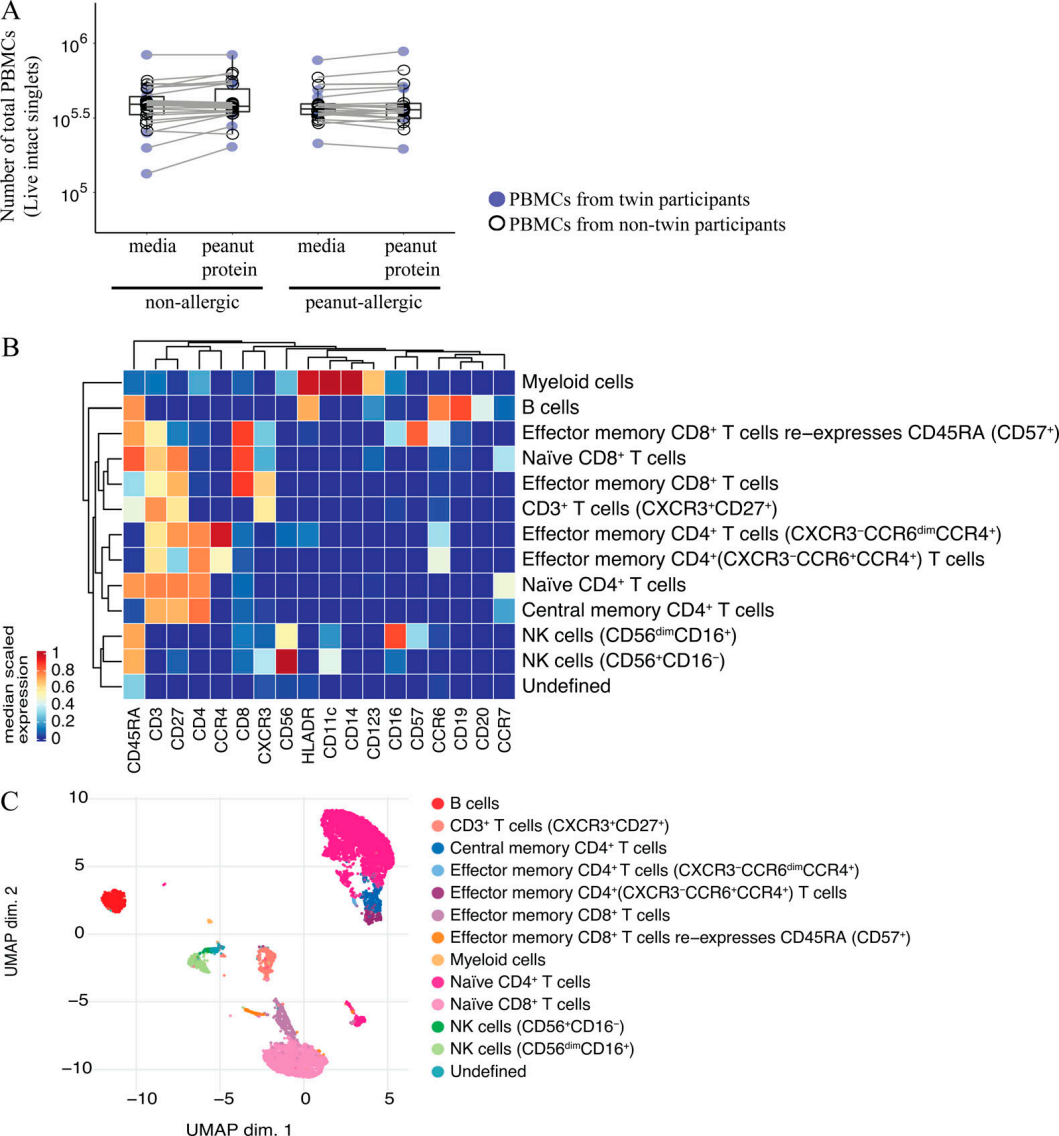
**No change in total live cell number when PBMCs are incubated with or without peanut protein for 3 d or when the subjects are NA or PA and the immune cell subsets are identified using unsupervised FlowSOM-based clustering analysis. (A) **The number of total live PBMCs stimulated with or without peanut protein in NA and PA participants. Blue circles represent samples from twin participants (*n* = 6 for each group); open circles represent the samples from nontwin participants (NA, *n* = 20; PA, *n* = 16). Each pair of points connected by a line represents one subject. Box plots represent the interquartile range (IQR) and median; whiskers extend to the farthest data point within a maximum of 1.5× IQR. Paired sample sets were analyzed using the Wilcoxon signed rank test (two sided). Unpaired sample sets were analyzed using the Wilcoxon rank sum test (two sided). **(B)** In each sample, 50,000 cells of the pregated live, single cells were randomly selected, and the marker expression values were inverse hyperbolic sine (Arcsinh)-transformed with a cofactor of 5. Heatmap representing the median expression levels of the marker in each cluster. 12 cell types were identified based on expression levels of markers in each cluster. **(C)** UMAP representation of 12,000 randomly selected cells (500 per file) with the identified cell types from the FlowSOM analysis.

To examine the difference in immune response to food antigen between allergic and NA individuals, we measured cytokine secretion using a Luminex-based assay. PBMCs from six pairs of twin siblings who were discordant for peanut allergy (i.e., one sibling has peanut allergy and the other is healthy without any allergies or other diseases) were incubated for 3 d either with or without peanut protein (Table S1). We chose twin pairs to minimize the confounding influence of genetic variation. Heatmap analysis showed that following peanut stimulation, supernatants from PBMCs secreted a greater number of cytokines in larger amounts in PA versus NA twin participants (Fig. 2 A). We compared the expression of 62 cytokines between the four experimental groups (NA vs. PA, each with or without peanut protein stimulation) using the Wilcoxon rank sum test and Wilcoxon signed rank test for unpaired and paired samples. In this analysis, a false discovery rate (FDR)–adjusted P value <0.1 was considered significant (Table S2). Because monocytes have been linked to food allergy (Neeland et al., 2018; Zhang et al., 2016), we presented the comparison results focused on 14 cytokines associated with monocyte activation in NA versus PA twin siblings with or without peanut protein stimulation (Arango Duque and Descoteaux, 2014; Bosch et al., 2002; Deshmane et al., 2009; Jenkins and Arend, 1993; Kaufmann et al., 1999; Norelli et al., 2018; Olszewski et al., 2000; Zhang et al., 2016). We found that compared with their NA siblings, peanut protein–stimulated PBMCs from PA twins showed significantly increased production of 10 cytokines (monocyte chemoattractant protein-1 [MCP1/CCL2], macrophage inflammatory protein 1α [MIP1A/CCL3], macrophage inflammatory protein 1 β [MIP1B/CCL4], IL-1 receptor antagonist [IL1RA], IL-1β, IL-6, TNFα, IL-10, IL12P70, and IL12P40; Fig. 2 B). The changes we observed were not indiscriminate, because we observed no difference in factors such as hepatocyte growth factor (HGF), leptin, or vascular cell adhesion molecule 1 (VCAM-1; Fig. 2 A). In addition, we observed no difference in the number of live cells between experimental conditions, suggesting that gross changes in cell death or survival do not underlie the changes in cytokine secretion (Fig. S1 A).

The distinct cytokine expression pattern of peanut-stimulated PBMCs from PA siblings was supported by an unsupervised principal component analysis (PCA) of the same 14 cytokines, which showed that the first and second principal components explained 75.6% of the variation within the dataset (Fig. 2 C). To confirm that this experimental group (i.e., PBMCs from PA siblings cultured with peanut protein) forms a distinct cluster, we performed a permutational ANOVA (PERMANOVA) for PCA (Skalski et al., 2018). This test examines the contribution of variables to the separation of the data in multiple dimensional space, and showed a significant P value among the groups (***, P < 0.001). Computation of Euclidean distances between the four groups also showed that the peanut-stimulated PBMCs from PA siblings behaved distinctly from the other groups (Fig. 2 D). These results show that peanut allergen stimulation results in the secretion of a set of monocyte-associated cytokines only in PA individuals.

### Changes in monocyte frequency and surface marker expression are associated with the allergen stimulation of PBMCs from PA children

**Figure S2.**
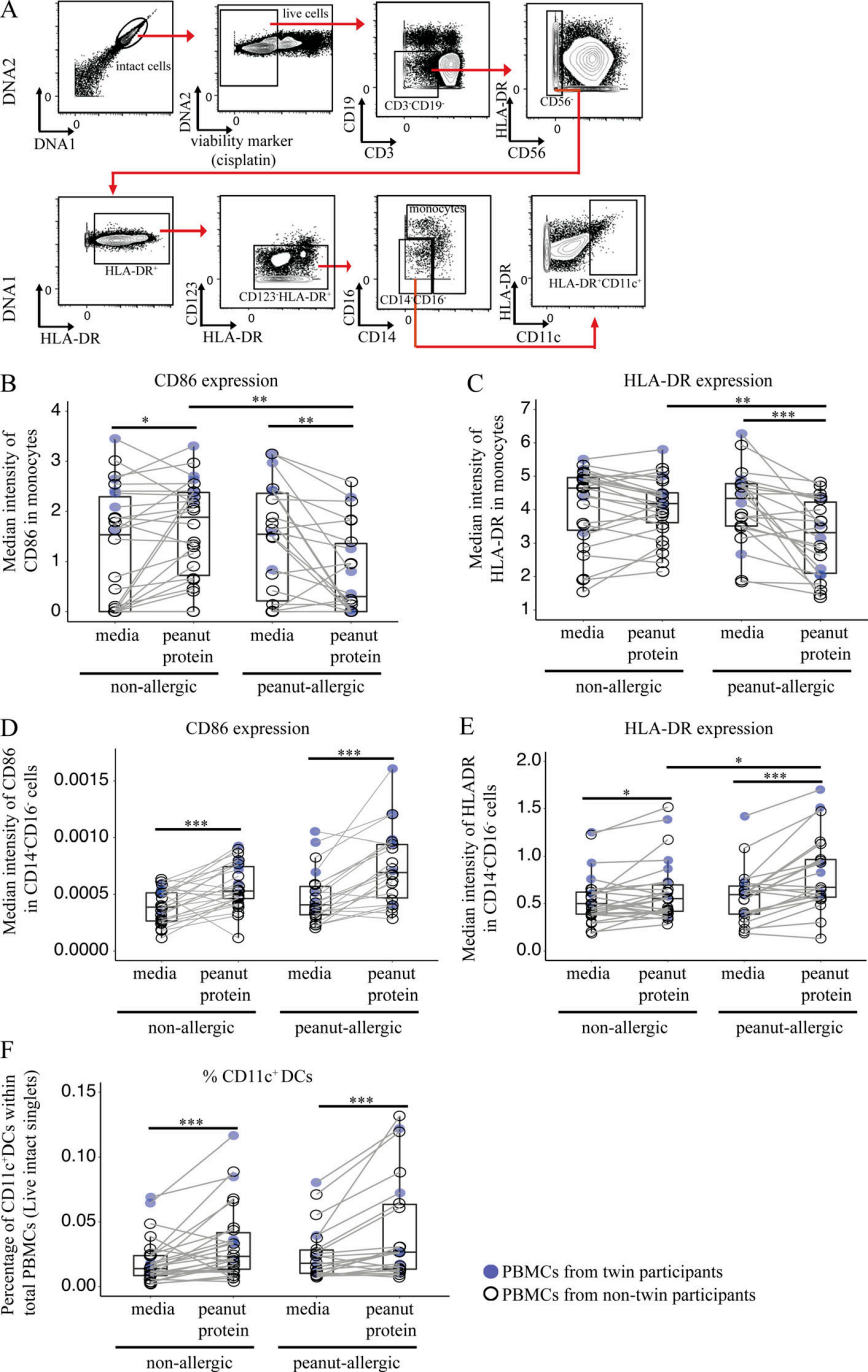
**Mass cytometric gating strategies for monocytes and CD11c^+^ DCs.** Incubation with peanut protein decreases the expression of CD86 and HLA-DR on monocytes for PA participants, increases CD86 and HLA-DR expression by Lin^−^CD14^−^CD16^−^HLA-DR^+^ cells for both NA and PA participants, and increases the percentage of CD11c^+^ DCs for both NA and PA participants. **(A)** Mass cytometric plots showing the gating strategies for monocytes and CD11c^+^ DCs. **(B and C)** The expression of CD86 (B) and HLA-DR (C) on monocytes from the PBMCs stimulated with or without peanut protein in NA and PA participants. **(D–F)** The expression of CD86 (D) and HLA-DR (E) on Lin (CD3, CD19, CD56, CD123)^−^HLA-DR^+^CD14^−^CD16^−^ cells and the percentage of CD11c^+^ DCs in PBMCs (C) in NA and PA participants stimulated with or without peanut protein. Box plots represent the interquartile range (IQR) and median; whiskers extend to the farthest data point within a maximum of 1.5× IQR. Blue circles represent samples from twin participants (*n* = 6 for each group); open circles represent the samples from nontwin participants (NA, *n* = 20; PA, *n* = 16). Each pair of points connected by a line represents one subject. Paired sample sets were analyzed using the Wilcoxon signed rank test (two sided). Unpaired sample sets were analyzed using the Wilcoxon rank sum test (two sided). *, P < 0.05; **, P < 0.01; ***, P < 0.001.

Given our cytokine results and previous reports associating monocytes with food allergy, we further analyzed monocytes by CyToF (Table S3, panel 1). We performed mass cytometry on PBMCs from the six twin pairs (same as those used in Luminex experiments) who are discordant for peanut allergy. PBMCs were again incubated with or without peanut protein for 3 d. We performed an unsupervised flow cytometry self-organizing maps (FlowSOM)–based clustering analysis for the PBMCs from the allergy-discordant twins, cultured with medium alone or medium plus peanut protein. This analysis identified 12 cell types based on cell surface expression markers (Fig. S1 B). We also performed a Uniform Manifold Approximation and Projection (UMAP) analysis to visualize this high-dimensional data. The cell clusters, which are color coded based on FlowSOM analysis, corresponded well to biological cell populations including T cells, B cells, natural killer cells, and the myeloid populations (Fig. S1 C). One disadvantage of unsupervised clustering is that populations of low abundance (<1%) are merged with larger populations bearing similar markers. Blood monocytes do not proliferate (Landsman and Jung, 2007; van Furth, 1989), and their frequency decreases during in vitro culture. As a result, the clustering algorithm merged the monocyte population with DCs. For this reason, we switched to manual gating to interrogate the monocyte population. Monocytes were identified as CD14^+^HLA-DR^+^ cells that were negative for the cell surface lineage markers CD3, CD19, CD56, and CD123. Based on their expression of CD14 and CD16, human monocytes were further subdivided as classic CD14^+^^+^CD16^−^, intermediate CD14^+^^+^CD16^+^, and nonclassic CD14^+^CD16^+^^+^ (Villani et al., 2017; Fig. S2 A).

**Figure 3.**
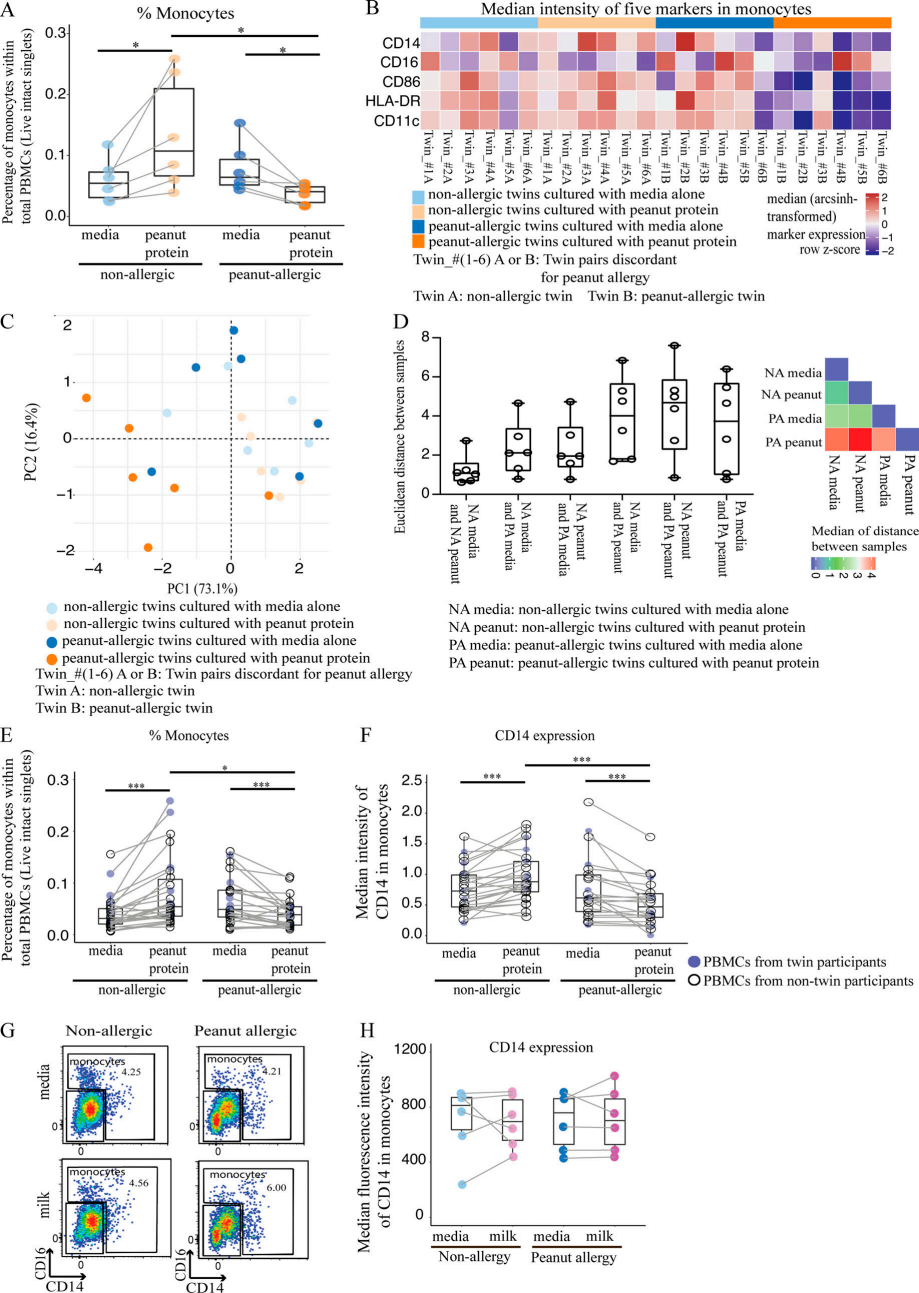
**Changes in monocyte frequency and surface markers are associated with the allergen stimulation of PBMCs from PA children. (A)** Monocyte percentages among PBMCs from six pairs of twin siblings discordant for peanut allergy. Each colored dot represents a single twin sample according to allergic status and culture conditions. **(B)** Heatmap representing median expression levels for CD14, CD16, CD86, HLA-DR, and CD11c on monocytes gated from PBMCs stimulated with or without peanut protein for six pairs of peanut allergy–discordant twins. Each column represents PBMCs from the indicated twin sibling cultured with medium alone or with peanut protein. Each row represents the normalized median expression using z-scores. Red and blue indicate higher and lower expression, respectively. Colored bars at the top of the heatmap indicate peanut allergy status and culture conditions for each experimental group. **(C)** PCA of five monocyte-related markers for six peanut allergy–discordant twin pairs. The percentage variances explained by principal component 1 (PC1) and PC2 are shown. Each point represents a single twin sample color-coded according to allergic status and peanut protein stimulation status. **(D)** Euclidean distances computed based on five monocyte-related markers for six pairs of peanut allergy–discordant twins. Box plots overlaid with dot plots represent the Euclidean distances for each pair of twin samples per each group (left), and the median values of distance for each group are displayed in a heatmap (right). **(E and F)** The percentage of monocytes among total PBMCs (E) and the median expression of CD14 on monocytes (F) from NA and PA samples stimulated with or without peanut protein. Blue circles represent samples from twin participants (*n* = 6 for each group); open circles represent the samples from nontwin participants (NA, *n* = 20; PA, *n* = 16). **(G and H)** Controls for the peanut allergen–specific down-regulation of CD14 expression on monocytes from PA subjects. Monocyte CD14 expression remained unchanged following PBMC incubation with cow’s milk for both PA and NA individuals who were not allergic to milk. Representative flow cytometry plots showing the monocyte population from PBMCs incubated with or without milk for one NA and one PA participant, neither allergic to milk (G). Monocyte CD14 expression after incubating PBMCs with or without milk for six NA and six PA participants who were not allergic to milk (H). Box plots in A, E, F, and H represent the interquartile range (IQR) and median; whiskers extend to the farthest data point within a maximum of 1.5× IQR. Paired sample sets were analyzed using the Wilcoxon signed rank test (two sided). Each pair of points connected by a line represents one subject. Unpaired sample sets were analyzed using the Wilcoxon rank sum test (two sided). *, P < 0.05; ***, P < 0.001.

We first quantified the effect of peanut stimulation on total monocyte frequency. Following peanut protein stimulation, the percentage of cells within the monocyte gate was significantly increased in NA siblings but significantly decreased in their paired PA twin siblings (Fig. 3 A). Because of the high degree of variability in the relative ratios of the classic/intermediate/nonclassic monocytes between individuals after cell culture, the differences in monocyte subpopulations between allergic and NA individuals were not analyzed further.

Next, we measured the expression levels of the monocyte-related markers CD14, CD16, CD86, HLA-DR, and CD11c in the cells within the monocyte gate. Heatmap analysis showed that peanut-stimulated monocytes from PA siblings presented a expression pattern distinct from the other three experimental groups (Fig. 3 B). A PCA performed using the same five markers showed that the first and second principal components accounted for 89.5% of the dataset variation and separated the majority of peanut-stimulated PA sibling samples from the other groups (Fig. 3 C). A PERMANOVA for PCA showed a significant P value among the groups (**, P < 0.01). A Euclidean distance–based analysis using the median expressions of five monocyte-related markers yielded a similar result (Fig. 3 D). These experiments suggest that monocytes from PA individuals manifest a distinct behavior, including decreased expression of CD14, CD86, and HLA-DR, after peanut protein stimulation.

To verify that our initial findings in twins were also valid in the general population, we tested PBMCs from 16 PA and 20 NA children who were age matched (Table S1). While the addition of peanut significantly increased the percentage of cells within the monocyte gate in NA participants, the opposite occurred following PBMC peanut stimulation in PA participants, in whom the percentage of monocytes decreased by 35% (Fig. 3 E; P = 0.0006). These results confirmed our original findings in twin siblings who were discordant for peanut allergy.

The decrease in frequency of cells within the monocyte gate in PA cultures could have occurred through cell death in vitro or through the transition of the monocytes into another cell subset. We observed no significant change in the number of live cells, regardless of whether PBMCs were incubated with or without peanut protein or whether the subjects were PA or NA (Fig. S1 A). This suggested that monocyte differentiation occurred, so we next examined the expression levels of monocyte-related markers individually to determine how they are affected by allergen stimulus.

We began with monocyte marker CD14. Exposure to farm animals has been linked to a decreased risk of developing atopic disease, as most recently reported by Stein et al. (2016). Along this vein, prior studies have demonstrated that the expression of CD14 transcript was significantly higher in the blood of children growing up on farms (Ege et al., 2006) or on the fetal side of placentas from pregnant mothers living on farms (Joerink et al., 2010). Therefore, we next used mass cytometry to directly compare CD14 protein expression on monocytes from PA and NA participants with or without peanut protein stimulation. After peanut stimulation, we observed a 26.7% increase in CD14 expression on monocytes from NA participants (P = 0.0002) versus a 30.6% decrease in CD14 expression on monocytes from PA participants (P = 0.0003; Fig. 3 F). Thus, monocytes from PA versus NA participants react very differently to allergen, with opposite responses in terms of cell frequency (shown above) and CD14 expression.

To determine whether the changes in CD14 expression between NA versus PA participants following peanut stimulation are allergen specific, we tested whether cow’s milk (another common food allergen) had a similar effect. We incubated PBMCs from PA (*n* = 6) and NA (*n* = 6) subjects either with or without cow’s milk protein for 3 d (Table S4). None of these individuals were allergic to milk. Following incubation with milk protein, CD14 expression on monocytes remained unchanged in both NA and PA participants (Fig. 3, G and H). We conclude that the changes in monocyte CD14 expression that we observed in PA subjects are allergen specific.

Because our data suggested that the monocyte response was specific to antigen (i.e., allergen), we then examined the expression levels of the costimulatory molecule, CD86, and the antigen presentation molecule, HLA-DR, on monocytes. The expression of CD86 and HLA-DR decreased significantly, by 44.43% (P = 0.0037) and 23.36% (P = 0.00003), respectively, on monocytes following peanut protein stimulation compared with culture medium alone in PA individuals (Fig. S2, B and C). In contrast, the expression of HLA-DR did not change, whereas that of CD86 increased by 28.6% (P = 0.0190) in NA individuals (Fig. S2, B and C). These results support our original findings from heatmap analysis of discordant twins showing that PBMCs from PA subjects respond differently to allergen, and raise the possibility that monocytes take a distinct pathway of differentiation in PA individuals.

### Peanut allergen promotes monocyte differentiation into CD209^+^ DCs in peanut allergy

We sought to understand the fate of monocytes following peanut protein stimulation in PA patients and hypothesized that they differentiate into other APCs. First, we examined the HLA-DR^+^CD14^−^CD16^−^ (Lin^−^) nonmonocyte population using mass cytometry (Fig. S2 A). The expression of HLA-DR and CD86 and the percentage of CD11c^+^ DCs were all significantly increased by peanut stimulation in PA patients (Fig. S2, D–F). However, this increase was statistically different from NA individuals only in the case of HLA-DR (41% in PA vs. 15% in NA; P = 0.0.036). Because CD11c^+^ DCs were increased in both allergic and NA subjects following peanut stimulation (71% and 75% in PA and NA participants, respectively), we shifted our attention to DC subsets.

**Figure 4.**
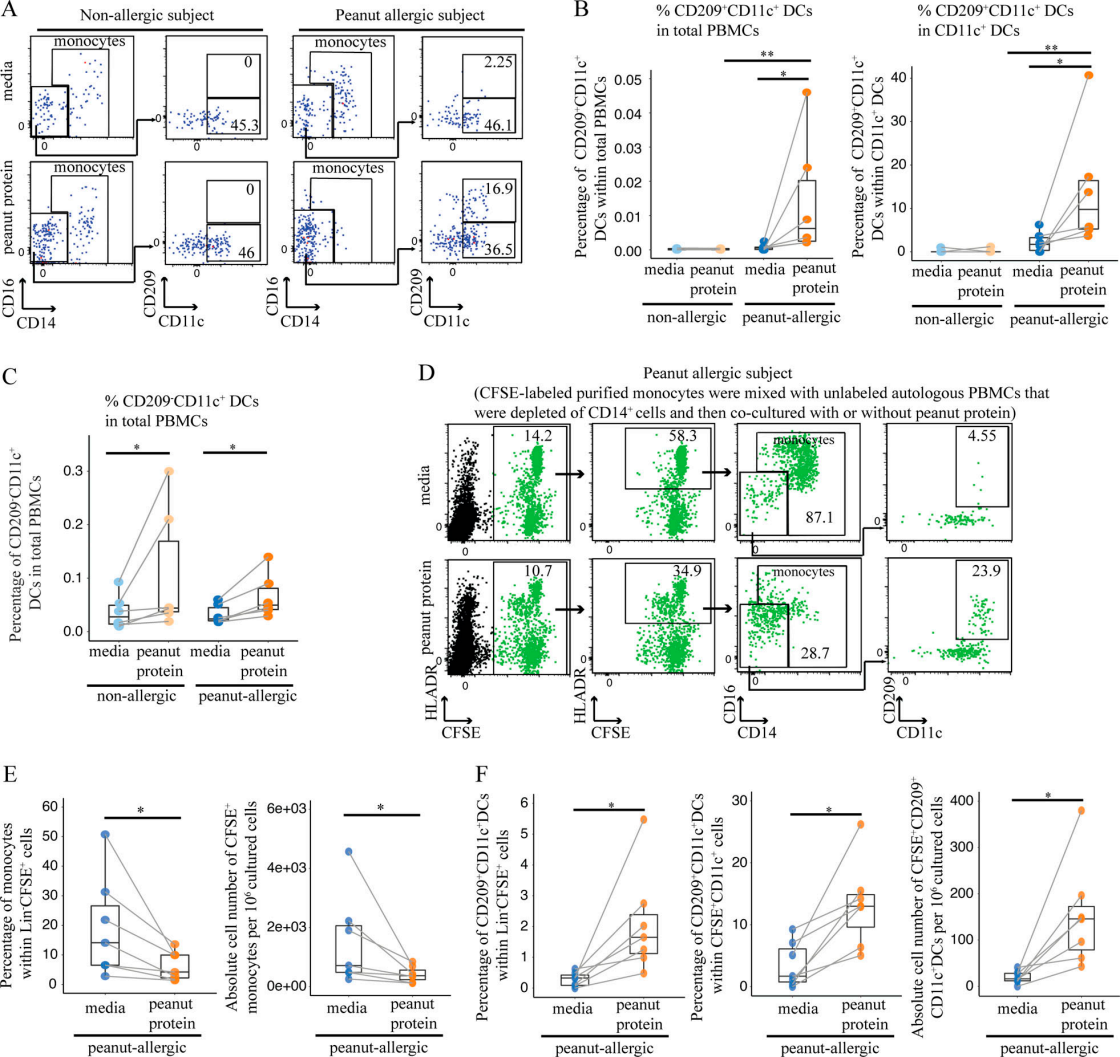
**Peanut allergen promotes monocyte differentiation into CD209^+^ DCs in peanut allergy. (A)** Representative flow cytometry plots gated on lineage (CD3, CD19, CD56) negative (Lin^−^) HLA-DR^+^ cells show the effect of peanut protein on CD209^+^CD11c^+^ and CD209^−^CD11c^+^ DCs for one NA and one PA nontwin participant. The frequencies of the gated populations are shown. **(B and C)** Percentage of CD209^+^CD11c^+^ DCs per total PBMCs or per CD11c^+^ DCs (B) and percentage of CD209^−^CD11c^+^ DCs per total PBMCs (C) are shown in box plots overlaid with dot plots from six NA and six PA nontwin participants. **(D)** Tracking monocyte differentiation by CFSE labeling. Representative flow cytometry plots gated on Lin^−^ cells show the effect of peanut protein on CFSE^+^ monocytes and CFSE^+^CD209^+^CD11c^+^ DCs for one PA nontwin participant. **(E)** The percentage of CFSE^+^ monocytes per Lin^−^CFSE^+^ cells (left) and their absolute number (right) per 10^6^ cultured cells are shown in box plots overlaid with dot plots for seven PA nontwin participants. **(F)** The percentage of CFSE^+^CD209^+^CD11c^+^ DCs per Lin^−^CFSE^+^ cells (left) or per Lin^−^CFSE^+^CD11c^+^ DCs (middle), as well as the absolute number of CFSE^+^CD209^+^CD11c^+^ DCs (right) per 10^6^ cultured cells, are shown in box plots overlaid with dot plots for seven PA nontwin participants. Box plots in B, C, E, and F represent IQR and median; whiskers extend to the farthest data point within a maximum of 1.5× IQR. Each pair of points connected by a line represents one subject. Paired sample sets were analyzed using the Wilcoxon signed rank test (two sided). Unpaired sample sets were analyzed using the Wilcoxon rank sum test (two sided). *, P < 0.05; **, P < 0.01.

CD209 (also called DC-specific intercellular adhesion molecule 3–grabbing nonintegrin, or DC-SIGN) is a unique marker, expressed on MDDCs but not on monocyte-derived macrophages or myeloid CD11c^+^ DCs (Osugi et al., 2002; Vento-Tormo et al., 2016). Interestingly, CD209 is involved in antigen capture, while MDDCs can skew CD4^+^ T cells toward Th2 responses (Cheong et al., 2010; Shreffler et al., 2006; Tanaka et al., 2000). Using flow cytometry, we quantified CD209^+^CD11c^+^ MDDCs following peanut protein stimulation and found this cell population to be significantly increased in PA, but not NA PBMCs (Fig. 4, A and B; and Table S5). CD11c^+^ DCs without CD209 were increased in both NA and PA participants after peanut stimulation (Fig. 4, A and C), similar to the previous result (Fig. S2 F).

**Figure S3.**
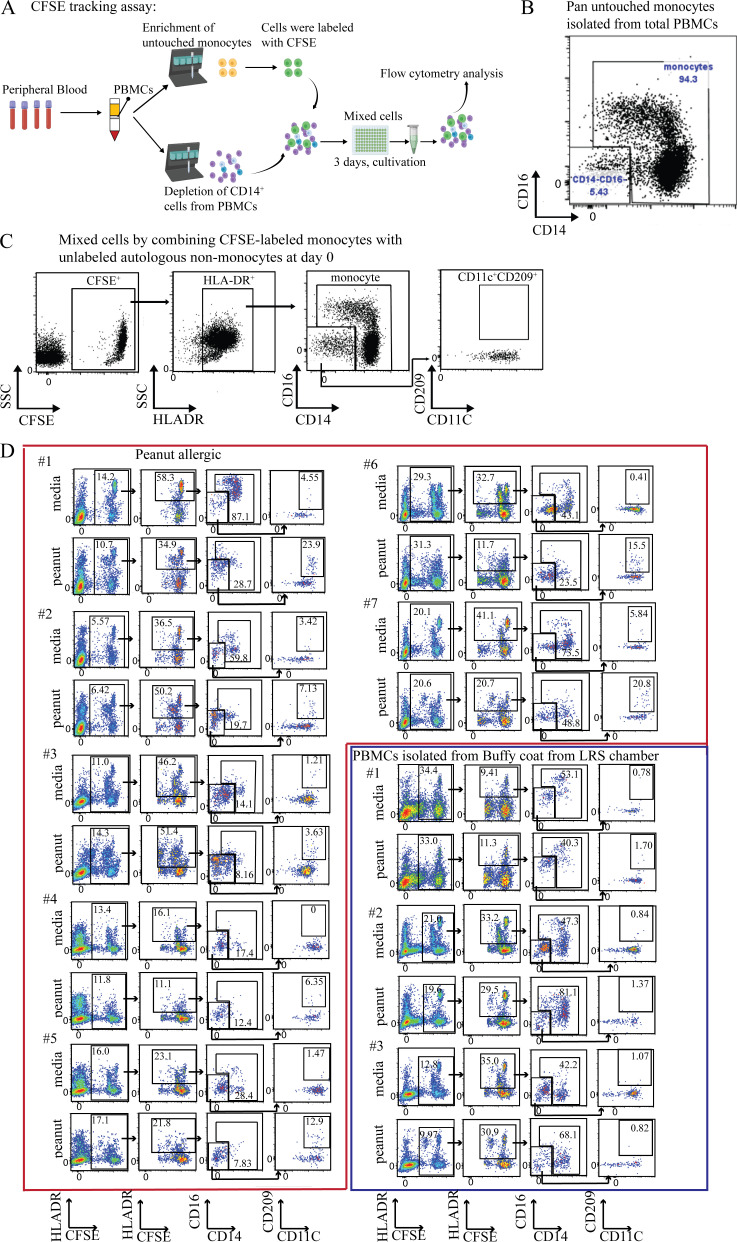
**Schematic overview and flow cytometry plots showing the CFSE-based assay for tracking monocyte changes in response to peanut protein for PA subjects. (A)** Schematic overview of the CFSE-based monocyte tracking assay. **(B)** Representative flow cytometry plots showing the purity of isolated untouched monocytes using Miltenyi pan monocytes isolation kit. **(C)** Representative flow cytometry plots show the CFSE^+^CD209^+^CD11c^+^ DCs in the mixed cells by combining CFSE-labeled monocytes and unlabeled autologous nonmonocytes before incubation with peanut proteins. **(D)** Flow cytometry plots gated on Lin (CD3, CD19, CD56) negative cells show the change of CFSE^+^ monocytes and CFSE^+^CD209^+^CD11c^+^ DCs by peanut protein from the PBMCs stimulated with or without peanut protein in seven PA participants and three healthy buffy coats.

To confirm that the CD209^+^CD11c^+^ cells we observed after peanut stimulation were derived from monocytes, we labeled purified monocytes from PA participants with the tracking dye CFSE (Fig. S3 A and Table S6). After pan purification, 95% of Lin^−^HLA-DR^+^ cells were verified as monocytes (Fig. S3 B), while none of the remaining small percentage of CD11c^+^ DCs expressed CD209 (Fig. S3 C). These CFSE-labeled monocytes were mixed with unlabeled autologous PBMCs that were depleted of CD14^+^ cells (i.e., monocytes) and then cocultured for 3 d with or without peanut protein (Fig. S3 A).

Consistent with our previous experiments, we found that the frequency and absolute number of CFSE^+^ monocytes decreased after peanut stimulation (Fig. 4, D and E; and Fig. S3 D), whereas the frequency and absolute number of CFSE^+^CD209^+^CD11c^+^ DCs increased (Fig. 4, D and F; and Fig. S3 D). Taken together, these data suggest that the CD209^+^ DCs that appear after peanut stimulation in PA participants are derived from monocytes.

### Peanut allergen augments CD23 expression on myeloid CD11c^+^ DCs

**Figure 5.**
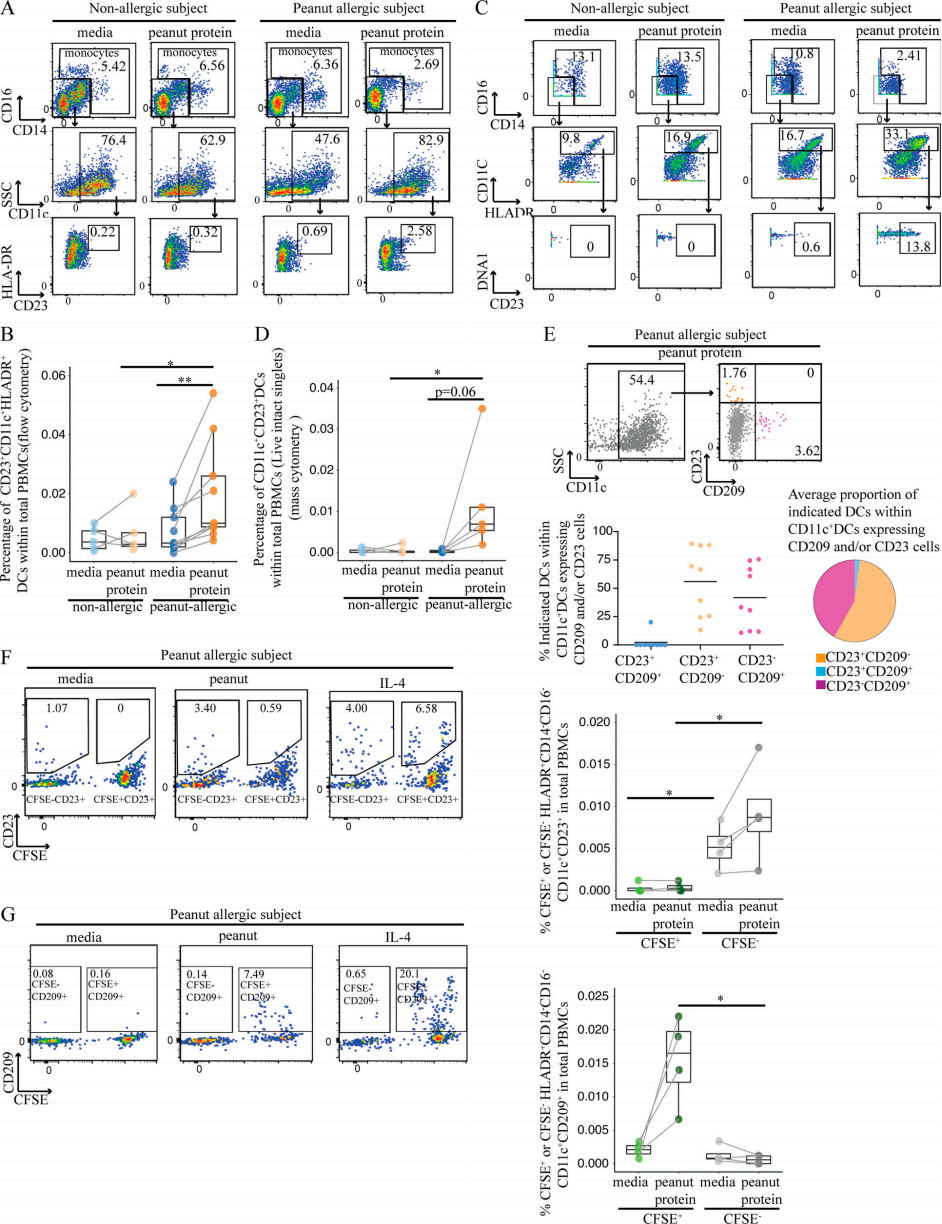
**Peanut allergen augments CD23 expression on myeloid CD11c^+^ DCs. (A)** Representative flow cytometry plots gated on lineage (CD3, CD19, CD56) negative (Lin^−^) HLA-DR^+^ cells show the effect of peanut protein on CD14^−^CD16^−^CD23^+^CD11c^+^ DCs for one NA and one PA nontwin participant. **(B)** Box plots overlaid with dot plots show the percentage of CD23^+^CD11c^+^ DCs per total PBMCs from five NA and nine PA nontwin participants with or without peanut stimulation. **(C)** Representative mass cytometry plots gated on lineage (CD3, CD19, CD56, CD123) negative (Lin^−^) HLA-DR^+^ cells show the effect of peanut protein on CD14^−^CD16^−^CD23^+^CD11c^+^ DCs for one NA and one PA nontwin participant. **(D)** Percentage CD23^+^CD11c^+^ DCs per total PBMCs incubated with or without peanut protein are shown in box plots overlaid with dot plots for five NA and five PA nontwin participants. **(E)** Top: Representative flow cytometry plots showing the effect of peanut protein on the expression of CD23 and CD209 by Lin^−^HLA-DR^+^CD14^−^CD16^−^CD11c^+^ DCs from one PA nontwin participant. The frequencies of the gated populations are shown. Bottom left: Distribution of CD209 and CD23 expression among CD11c^+^ DCs that expressed one or both markers. Bottom right: Among CD11c^+^ DCs that expressed CD209 and/or CD23, pie charts showing the average proportion of cells expressing one or the other marker. PBMCs from PA individuals were incubated with peanut protein (*n* = 9). **(F and G)** Left: Representative flow cytometry plots gated on Lin^−^HLA-DR^+^CD14^−^CD16^−^CD11c^+^ DCs show the expression of CD23 (F) or CD209 (G) on CFSE^−^ or CFSE^+^ cells for one PA nontwin participant. The cells from the same subject stimulated with IL-4 for 3 d are evaluated as the positive control for the expression of CD23 or CD209 on CFSE^+^ or CFSE^−^ cells. Right: Percentage of CD23^+^ (F) or CD209^+^ (G) DCs segregated by CFSE staining for four PA nontwin participants. Box plots in B, D, F, and G represent IQR and median; whiskers extend to the farthest data point within a maximum of 1.5× IQR. Paired sample sets were analyzed using the Wilcoxon signed rank test (two sided). Each pair of points connected by a line represents one subject. Unpaired sample sets were analyzed using the Wilcoxon rank sum test (two sided). *, P < 0.05; **, P < 0.01.

**Figure S4.**
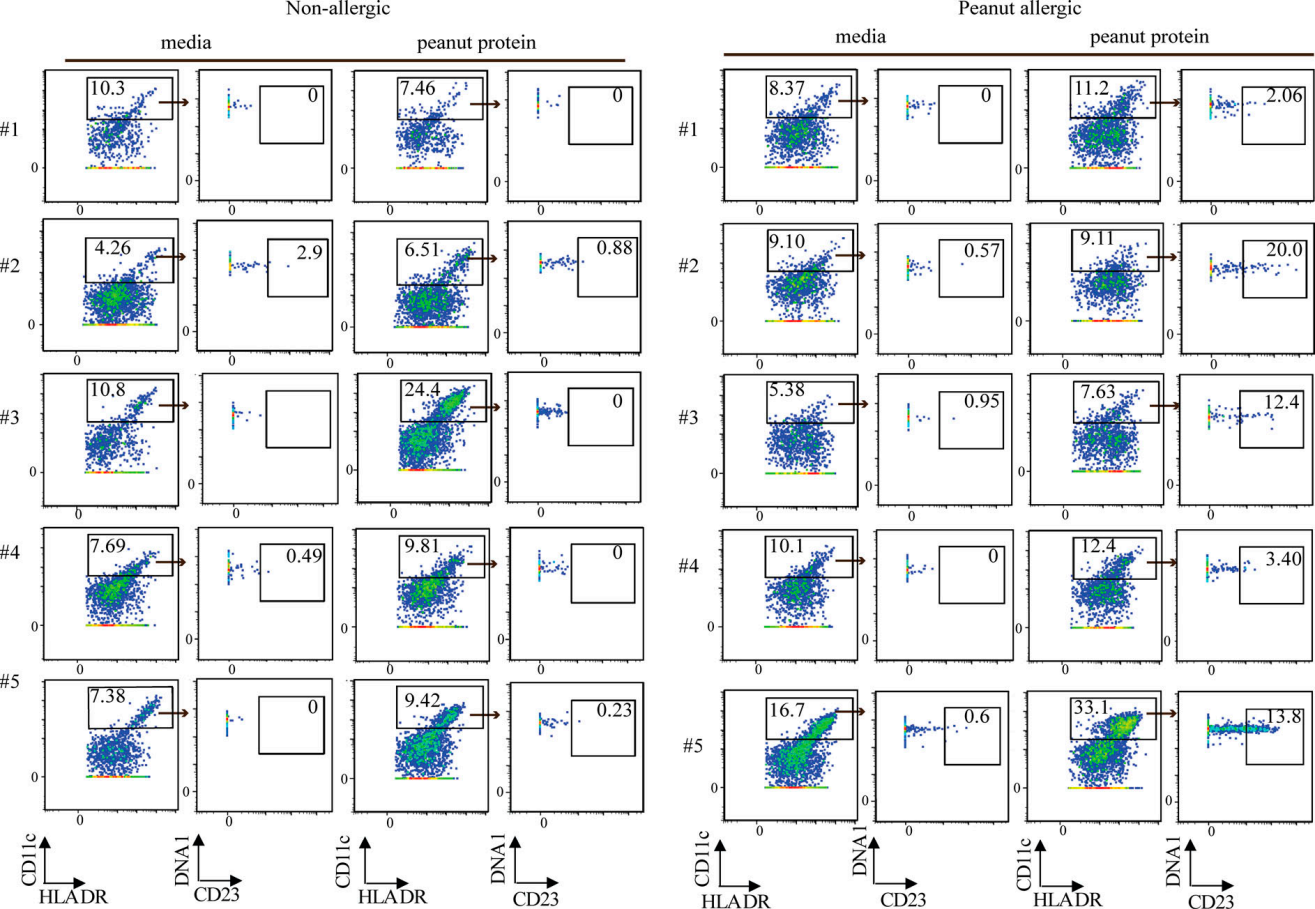
**CD23^+^CD11c^+^ DCs are induced by peanut protein in PA, but not NA, participants.** Mass cytometry plots gated on Lin (CD3, CD19, CD56, CD123)^−^HLA-DR^+^CD14^−^CD16^−^ cells showing the change of CD23^+^CD11c^+^ DCs by peanut protein for five NA and five PA nontwin participants.

In looking for other patterns of APC differentiation in food allergy, we examined the expression of the low-affinity IgE receptor CD23, which plays an important role in regulating IgE production and IgE-mediated immune and inflammatory functions in food allergy (Johnston et al., 2014), and the expression of which is traditionally associated with B cells (Acharya et al., 2010). We measured the expression of CD23 among DCs from PA and NA participants with or without peanut protein stimulation. We observed a striking increase in the frequency of CD23^+^CD11c^+^ DCs following peanut stimulation in PA participants compared with NA participants in two independent experimental sets using flow cytometry (Fig. 5, A and B; and Table S7) or mass cytometry (Fig. 5, C and D; Fig. S4; Table S3, panel 2; and Table S8).

We measured the proportion of DCs expressing CD23, CD209, or both to determine if CD23 and CD209 were coexpressed on DCs (Table S7). Interestingly, the expression of CD23 and CD209 was, for the most part, mutually exclusive on CD11c^+^ DCs after peanut stimulation of PA-derived PBMCs (Fig. 5 E). This suggested that separate populations of DCs were expressing CD23 versus CD209.

To further dissect whether CD23 and CD209 expression defined separate DC populations, we returned to the previous experimental system, where CFSE-labeled, purified monocytes were mixed with unlabeled autologous nonmonocyte PBMCs that were depleted of CD14^+^ cells (Fig. S3 A and Table S9). After culturing PA PBMCs with or without peanut protein for 3 d, we observed that CD23 was expressed primarily on CFSE-negative DCs (Fig. 5 F). In contrast, CD209 was expressed on CFSE^+^ DCs derived from monocytes (Fig. 5 G). These results indicate that myeloid CD11c^+^ DCs express CD23, whereas MDDCs express CD209, after peanut stimulation of PBMCs in PA individuals. Because CD209 promotes the uptake of food antigen for processing and presentation by APCs, while CD23 captures (food antigen–specific) IgE, we speculate that these separate DC subsets perform distinct roles in IgE-mediated allergy.

### Th2 cytokines and signaling through IL4RA are responsible for the expression of CD209 and CD23 by DCs after peanut allergen stimulation

**Figure S5.**
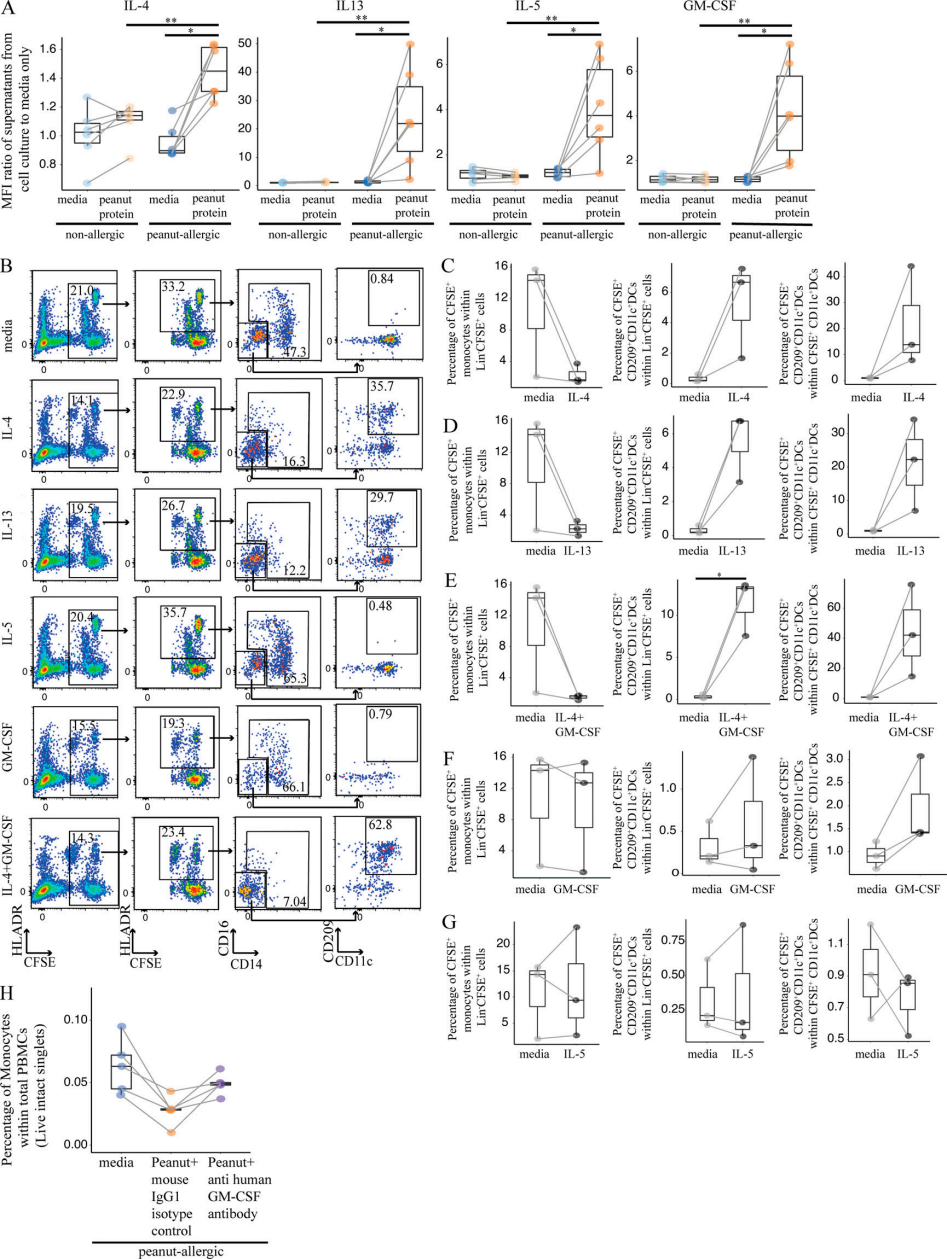
**Th2 cytokines promote monocyte differentiation into CD209^+^ DCs, and blocking GM-CSF partially reverses the decrease in monocyte frequency induced by peanut protein in PA participants. (A)** The expression levels of secreted Th2 cytokines from the PBMCs cultured with or without peanut protein from six pairs of discordant twin siblings for peanut allergy. Each dot represents a single participant, colored according to different treatment. Each pair of points connected by a line represents one subject. **(B)** Representative flow cytometry plots gated on lineage (CD3, CD19, CD56) negative cells show the change of CFSE^+^ monocytes and CFSE^+^CD209^+^CD11C^+^ DCs by IL-4, IL-13, IL-5, GM-CSF, or IL4 + GM-CSF from buffy coats. **(C–G)** Percentages of CFSE^+^ monocytes in Lin^−^CFSE^+^ cells (left) and of CFSE^+^CD209^+^CD11c^+^ DCs in Lin^−^CFSE^+^ cells (middle) or in CFSE^+^CD11c^+^ DCs (right) from the PBMCs treated with IL-4 (C), IL-13 (D), IL4 + GM-CSF (E), GM-CSF (F), or IL-5 (G) from three buffy coats. **(H)** Percentages of monocytes in PBMCs are shown in box plots overlaid with dot plots from five PA participants treated with peanut protein + anti-GM-CSF antibody and peanut protein + isotype control for anti-GM-CSF antibody (mouse IgG1). Box plots in A and C–H represent the interquartile range (IQR) and median; whiskers extend to the farthest data point within a maximum of 1.5× IQR. Each pair of points connected by a line represents one subject. Paired sample sets were analyzed using the Wilcoxon signed rank test (two sided). Unpaired sample sets were analyzed using the Wilcoxon rank sum test (two sided). *, P < 0.05; **, P < 0.01.

Prior studies have suggested that the expression of either CD209 or CD23 on DC is dependent on IL-4 (Poole et al., 2005; Relloso et al., 2002; Sander et al., 2017; Williams et al., 1992). Consistent with this possibility, we detected significantly more secreted IL-4 as well as IL-13, IL-5, and GM-CSF following peanut stimulation of PA PBMCs compared with NA PBMCs in Luminex assays (Fig. S5 A).

We performed a series of experiments to test whether these cytokines alone could induce the same changes in monocytes from NA individuals that we had observed in PBMCs from allergic participants after peanut protein stimulation. CFSE-labeled monocytes were isolated from healthy blood donor buffy coats and mixed with unlabeled nonmonocyte PBMCs as before (Fig. S3 A). These mixed cells were then cultured with medium alone, IL-4, IL-13, IL-5, or GM-CSF for 3 d. Stimulation with both GM-CSF and IL-4 was included as a positive control, as this combination of cytokines had been shown to promote monocyte differentiation toward DCs in vitro (Sander et al., 2017). We then evaluated the extent of monocyte differentiation by measuring the change in monocyte frequency and the appearance of CFSE^+^CD209^+^CD11c^+^ DCs after cytokine stimulation.

After 3-d culture, we found that either IL-4 or IL-13 decreased the percentage of monocytes and increased the proportion of CD209^+^CD11c^+^ DCs, similar to the changes we observed in PBMCs from PA participants (Fig. S5, B–G). These changes were most pronounced after stimulation with IL-4 + GM-CSF, while no effect was seen with IL-5. These results suggested that IL-4 and IL-13 were responsible for the APC differentiation that occurs in PA PBMCs after allergen stimulation, with a possible contribution from GM-CSF.

To determine whether IL-4 and IL-13 are necessary for the APC differentiation, we added a monoclonal blocking antibody against IL4RA to peanut-stimulated PBMCs from PA participants (Table S6). IL4RA is part of both the IL-4 and IL-13 receptors, and its blockade inhibits signaling by both cytokines. Monocytes were labeled with CFSE as before to facilitate tracking of their differentiation.

**Figure 6.**
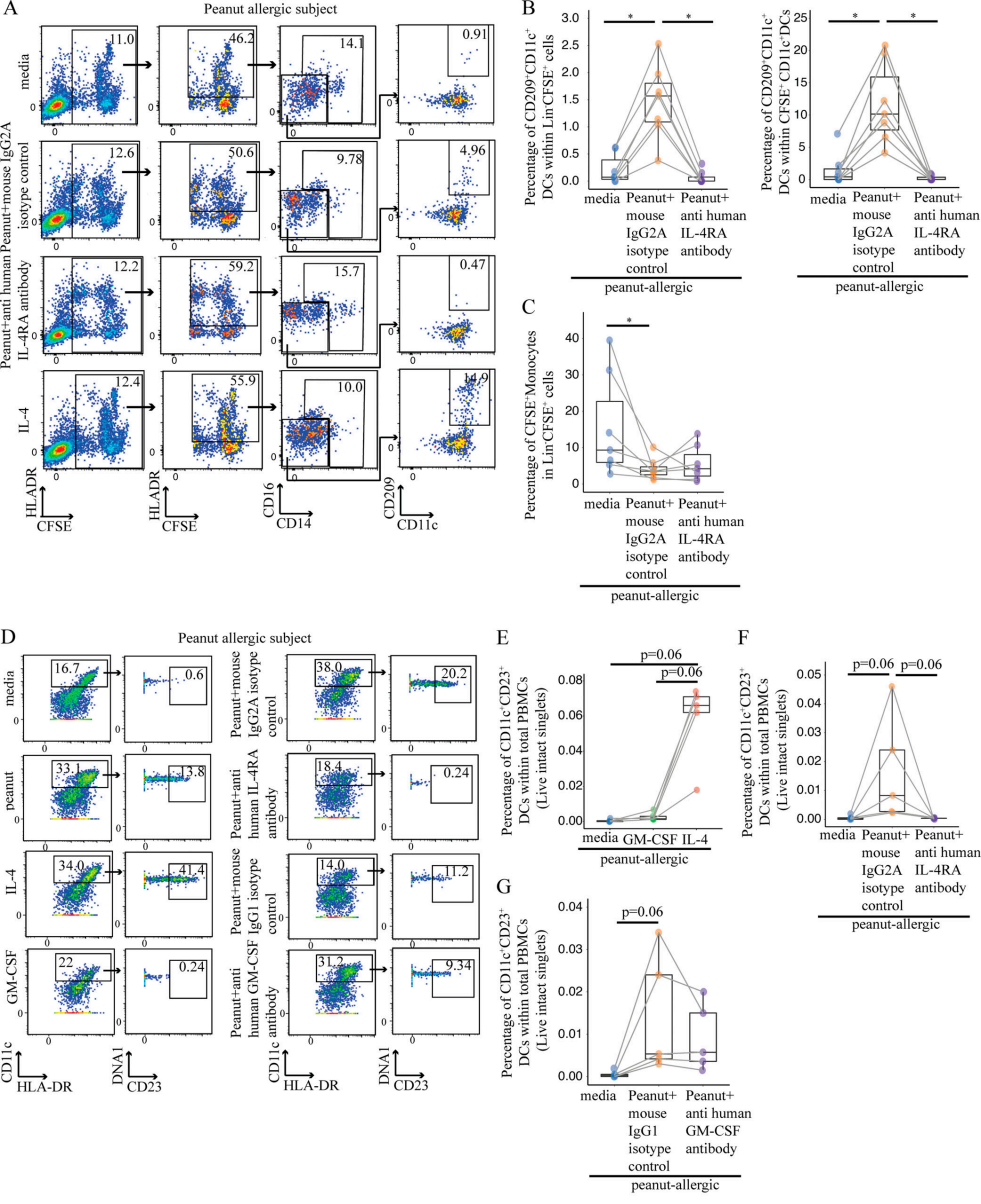
**Signaling through IL4RA is responsible for the expression of CD209 and CD23 by DCs after peanut allergen stimulation. (A)** Representative flow cytometry plots gated on Lin^−^ cells show the effect of peanut protein, anti-IL4RA blocking antibody, and IL-4 on CFSE-labeled monocytes and CD209^+^CD11c^+^ DCs for one PA nontwin participant. **(B and C)** The percentage of CFSE^+^CD209^+^CD11c^+^ DCs per Lin^−^CFSE^+^ cells (B, left) or Lin^−^CFSE^+^CD11c^+^ DCs (B, right), as well as the percentage of CFSE^+^ monocytes per Lin^−^CFSE^+^ cells (C), are shown for seven PA nontwin participants. **(D)** Representative mass cytometry plots gated on Lin^−^HLA-DR^+^CD14^−^CD16^−^ cells show the effects of peanut protein, IL-4, GM-CSF, and blocking antibodies to IL-4 or GM-CSF on CD23^+^CD11c^+^ DCs for one PA nontwin participant. **(E–G)** Percentages of CD23^+^CD11c^+^ DCs per total PBMCs from five PA nontwin participants treated with different combinations of peanut protein, IL-4, GM-CSF, and blocking antibodies against IL-4RA or GM-CSF. Box plots in B, C, and E–G represent IQR and median; whiskers extend to the farthest data point within a maximum of 1.5× IQR. Each pair of points connected by a line represents one subject. Paired sample sets were analyzed using the Wilcoxon signed rank test (two sided). Unpaired sample sets were analyzed using the Wilcoxon rank sum test (two sided). For the Wilcoxon signed rank comparisons, the lowest possible P value attainable for our analysis with five PA individuals is 0.0625. *, P < 0.05.

We found that anti-IL4RA antibody blocked the differentiation of monocytes into CD209^+^CD11c^+^ DCs, demonstrating that this process requires IL-4 and/or IL-13 (Fig. 6, A and B). Blocking IL4RA failed to reverse the decrease in monocyte percentage (Fig. 6 C), possibly due to the action of other cytokines released after peanut stimulation, including GM-CSF (Fig. 2 A and Fig. S5 A). Consistent with this, blocking GM-CSF showed a trend (*n* = 5, P = 0.0625) toward partial rescue of the decrease in monocyte frequency induced by peanut protein in PA participants (Fig. S5 H).

We also examined the effect of IL-4 and GM-CSF on CD23 expression by CD11c^+^ DCs. To determine if cytokines could replicate the effect of peanut protein on CD23 expression, PBMCs from PA patients were incubated for 3 d with medium, peanut protein, IL-4, or GM-CSF and then analyzed by mass cytometry. Stimulation with either peanut protein or IL-4 increased the percentage of CD23^+^CD11c^+^ DCs, while GM-CSF had no effect (Fig. 6, D and E; and Table S8). Conversely, to determine if blocking the same cytokines counteracted the effect of peanut protein on CD23 expression, PBMCs from PA participants were incubated with peanut protein as well as antibodies against IL4RA, GM-CSF, or the corresponding isotype controls (Table S8). PBMCs from NA controls were not used, because no induction of CD23 had been observed (Fig. 5, A–D; and Fig. S4). Blocking IL4RA (Fig. 6, D and F), but not GM-CSF (Fig. 6, D and G), inhibited the peanut-induced increase in CD23 expression by CD11c^+^ DCs. Thus, the ability of peanut protein to increase CD23 and CD209 expression on separate CD11c^+^ DC populations from PA participants is dependent on signaling through IL4RA.

### T cells are the major source of IL-4 and IL-13 responsible for monocyte and DC differentiation following peanut protein stimulation

**Figure 7.**
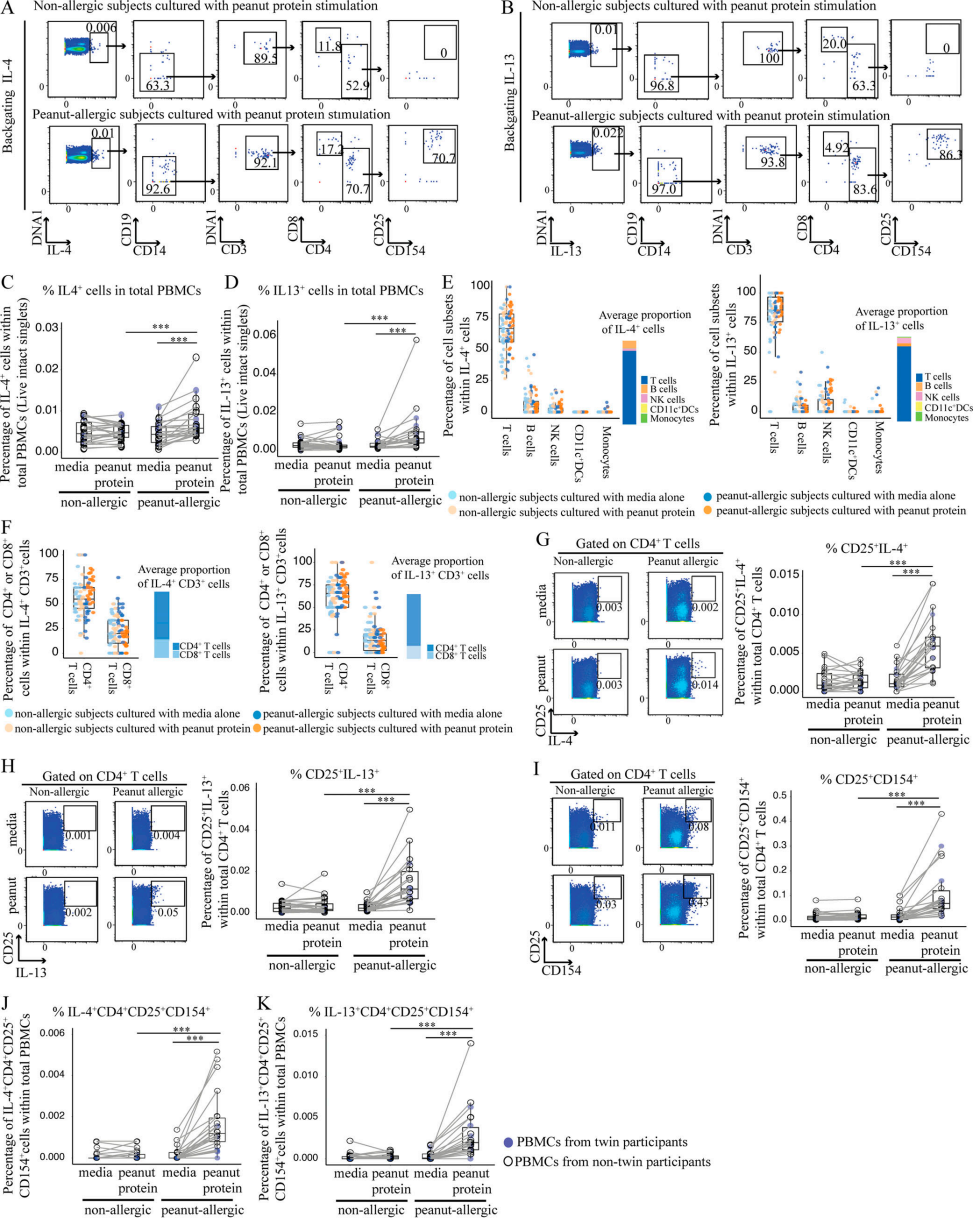
**T cells are the major source of IL-4 and IL-13 responsible for monocyte and DC differentiation following peanut protein stimulation. (A and B)** Representative mass cytometry plots showing CD4^+^ activated T cells as measured by CD25 and CD154 expression, identified by backgating from IL4^+^ cells (A) and IL-13^+^ cells (B) for one NA and one PA participant. **(C and D)** Percentage of IL-4^+^ (C) and IL-13^+^ (D) cells per total PBMCs incubated with or without peanut protein for NA versus PA participants. **(E and F)** Percentage of different cell types (T cells, B cells, natural killer cells, CD11c^+^ DCs, and monocytes) in IL-4^+^ cells (left) or IL-13^+^ cells (right; E). Percentage of CD4^+^ and CD8^+^ T cells in IL-4^+^CD3^+^ T cells (left) or IL-13^+^CD3^+^ T cells (right; F). Each dot represents a single subject color-coded according to allergic status and peanut protein stimulation status. NK, natural killer. **(G–I)** Left: Representative mass cytometry plots show the effect of peanut protein on CD4^+^ T cells for the expression of CD25 and IL-4 (G), IL-13 (H), or CD154 (I) for one NA and one PA participant. Right: Percentage of CD25^+^IL-4^+^ (G), CD25^+^IL-13^+^ (H), and CD25^+^CD154^+^ (I) CD4^+^ T cells for PBMCs from NA and PA participants incubated with or without peanut protein. **(J and K)** Mass cytometry backgating based on IL-4 and IL-13: Percentage of IL-4^+^CD25^+^CD154^+^CD4^+^ T cells (J) and IL-13^+^CD25^+^CD154^+^CD4^+^ T cells (K) per total PBMCs incubated with or without peanut protein for NA versus PA participants. Box plots in C, D, and G–K represent IQR and median; whiskers extend to the farthest data point within a maximum of 1.5× IQR. Blue circles represent twin participants (*n* = 6 for each group); open circles represent nontwin participants (NA, *n* = 20; PA, *n* = 16). Each pair of points connected by a line represents one subject. Paired sample sets were analyzed using the Wilcoxon signed rank test (two sided). Unpaired sample sets were analyzed using the Wilcoxon rank sum test (two sided). ***, P < 0.001.

To determine the cytokine source of IL4RA signaling following peanut stimulation of PBMCs from PA participants, we next performed backgating of IL-4^+^ cells and IL-13^+^ cells to identify the major IL-4– and IL-13–producing cell types (Fig. 7, A and B). As expected, we found that the frequency of IL-4^+^ cells and IL-13^+^ cells in total PBMCs was significantly increased in PA participants following peanut protein stimulation, but not in NA participants (Fig. 7, C and D). Backgating showed that CD4^+^ T cells constituted the majority of IL-4– and IL-13–producing cells (Fig. 7, E and F), although we note that basophils and eosinophils, which also secrete these cytokines, do not survive the freezing process used to bank the PBMC samples used in these experiments (Stone et al., 2010). Further analysis demonstrated that, following peanut protein stimulation, CD4^+^ T cells expressed significantly more IL-4 and IL-13, and the frequency of CD4^+^ activated T cells (CD4^+^CD25^+^CD154^+^) significantly increased in PBMCs from PA participants in comparison to NA participants (Fig. 7, G–I). In addition, a greater proportion of IL-4^+^CD4^+^ and IL-13^+^CD4^+^ T cells were activated, as measured by CD25 (IL-2RA) and CD154 (CD40L) expression, in PA PBMCs after peanut stimulation (Fig. 7, J and K). These results suggest that CD4^+^ T cells were the major IL-4– and IL-13–producing cell types in our experimental system.

**Figure 8.**
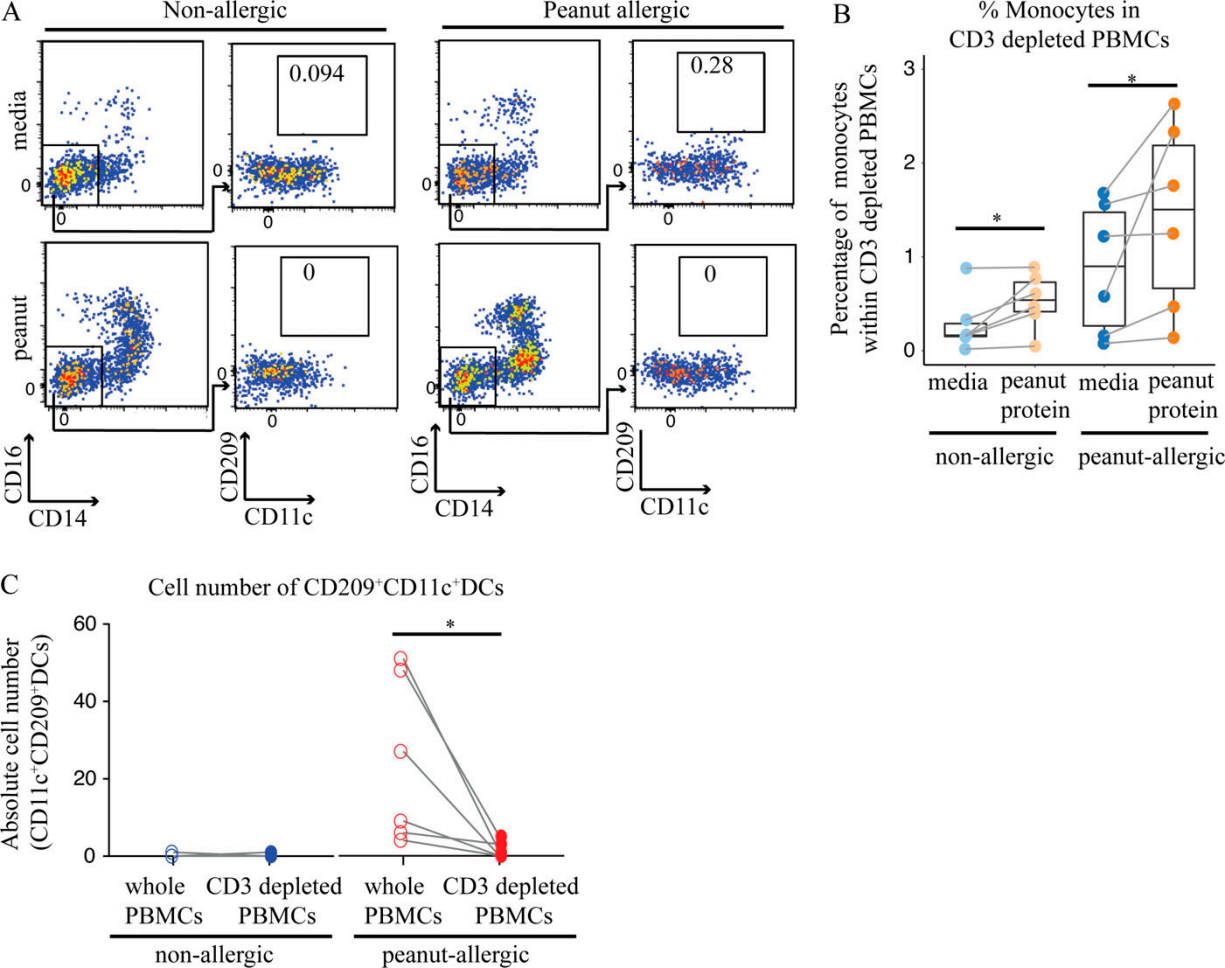
**Removing CD3^+^ cells from PA participants resulted in an increase in monocyte percentage and an absence of CD209^+^ MDDCs after peanut stimulation. (A)** Flow cytometry plots showing an increase in monocyte percentage and the absence of CD209^+^ MDDCs following peanut stimulation of CD3-depleted PBMCs from NA and PA participants. Flow cytometry plots gated on Lin^−^HLA-DR^+^ cells show the effect of CD3 depletion on CD209^+^CD11c^+^ DCs for one NA and one PA nontwin participant. **(B)** Box plots overlaid with dot plot show the percentage of monocytes in CD3-depleted PBMCs incubated with or without peanut protein for six NA and six PA nontwin participants. **(C)** Absolute number of CD209^+^CD11c^+^ DCs in either whole or CD3-depleted PBMCs incubated with peanut protein for six NA and six PA nontwin participants. Box plots in B represent IQR and median, and whiskers extend to the farthest data point within a maximum of 1.5× IQR. Each pair of points connected by a line represents one subject. Paired sample sets were analyzed using the Wilcoxon signed rank test (two sided). Unpaired sample sets were analyzed using the Wilcoxon rank sum test (two sided). *, P < 0.05.

To determine the importance of IL-4– and IL-13–producing T cells for APC differentiation in PA patients, we depleted CD3^+^ cells from the PBMCs of six PA and six NA participants before incubation with or without peanut protein for 3 d (Table S5). Whereas nondepleted PBMCs from PA participants showed a decrease in monocyte percentage and increase in CD209^+^ MDDCs following peanut stimulation, removing CD3^+^ cells resulted in the opposite: an increase in monocyte percentage and an absence of CD209^+^ MDDCs after peanut stimulation (Fig. 8, A–C). We conclude that IL-4– and/or IL-13–producing CD4^+^ T cells contribute to the differentiation of monocytes to CD209^+^ DCs.

### CD209^+^ DCs reinforce the Th2 cell response following peanut allergen stimulation

**Figure 9.**
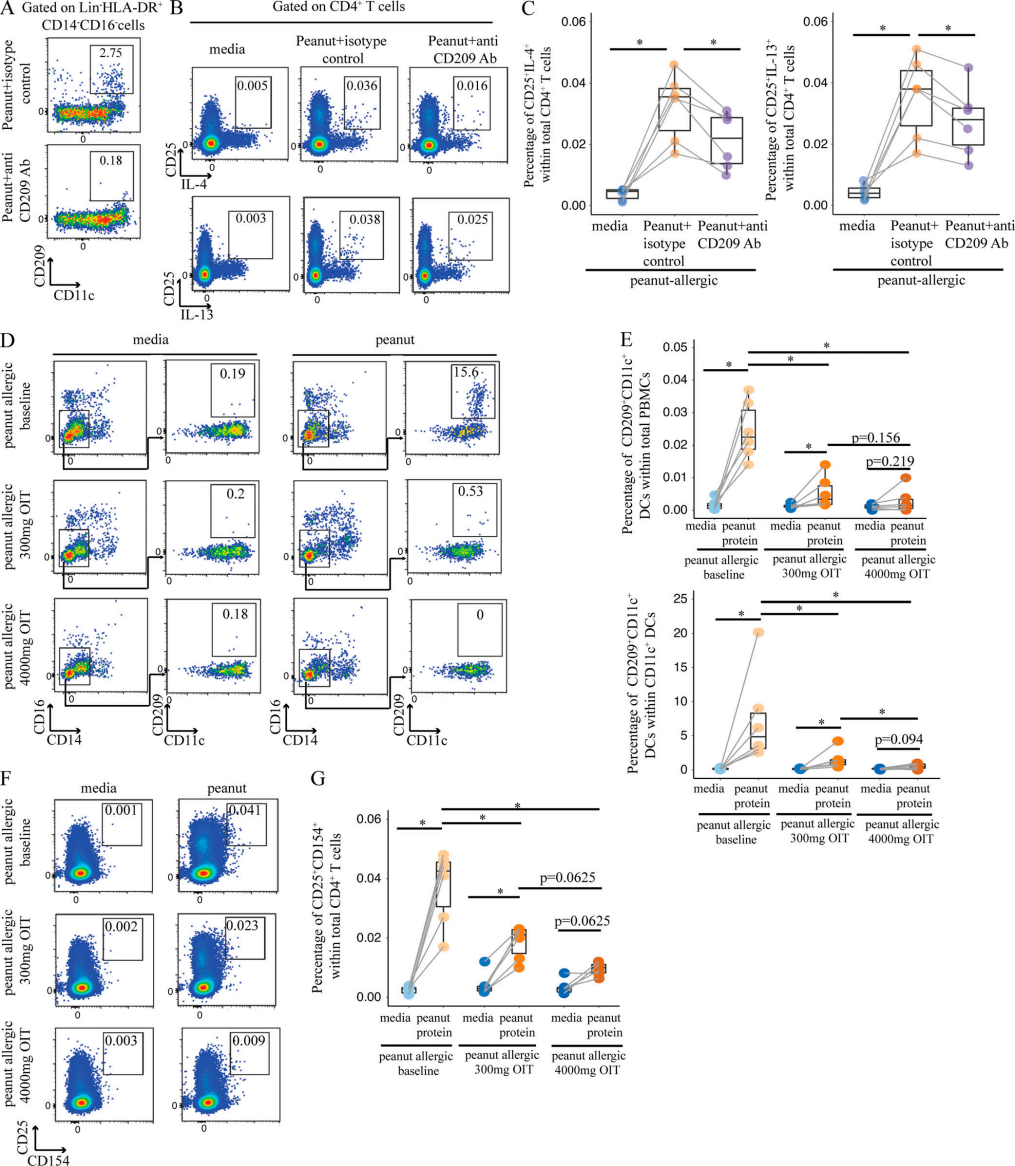
**CD209 promotes Th2 cytokine expression by peanut-specific T cells and correlates negatively with peanut OIT. (A–C)** Blocking CD209 expression in CD11c^+^ DCs reduces the production of Th2 cytokines (IL-4 and IL-13) from peanut-activated CD4^+^ T cells by peanut protein in PA participants. Representative flow cytometry plots gated on lineage (CD3, CD19, CD56) negative (Lin^−^) HLA-DR^+^CD14^−^CD16^−^ cells show the effect of anti-CD209 blocking antibody on CD209^+^CD11c^+^ DCs for one PA nontwin participant (A). Representative flow cytometry plots gated on CD4^+^ T cells show the effect of anti-CD209 blocking antibody on the expression of CD25, IL-4, and IL-13 for one PA nontwin participant (B). Percentage of CD4^+^ T cells expressing CD25 and either IL-4 (left) or IL-13 (right) for six PA nontwin participants (C). **(D–G)** The induction of CD209^+^CD11c^+^ DCs and peanut-specific CD4^+^ T cells after 3-d incubation with peanut protein is reduced by OIT. Representative flow cytometry plots gated on lineage (CD3, CD19, CD56) negative (Lin^−^) HLA-DR^+^ cells showing the effect of OIT on CD209^+^CD11c^+^ DCs for one PA participant (D). Percentage of CD209^+^CD11c^+^ DCs per total PBMCs (top) and per CD11c^+^ DCs (bottom) are shown for six PA participants before and during OIT (E). Representative flow cytometry plots gated on CD4^+^ T cells show the effect of OIT on expression of the activation markers CD25 and CD154 for one PA participant (F). Percentage of CD25^+^CD154^+^ cells per total CD4^+^ cells for six PA subjects before and during OIT (G). Box plots in 9, C, E, and G represent IQR and median; whiskers extend to the farthest data point within a maximum of 1.5× IQR. Each pair of points connected by a line represents one subject. Paired sample sets were analyzed using the Wilcoxon signed rank test (two sided). Unpaired sample sets were analyzed using the Wilcoxon rank sum test (two sided). *, P < 0.05.

Because CD209^+^ DCs have been reported to skew immune responses toward Th2 (Alonso et al., 2011), and CD209 itself has been reported to capture the peanut protein Ara h 1 (Shreffler et al., 2006), we tested whether the addition of anti-CD209 blocking monoclonal antibodies (Geijtenbeek et al., 2000; Shreffler et al., 2006) would affect the strength of the Th2 response we observed in our experimental system. PBMCs from PA individuals (Table S10) were incubated with peanut protein in the presence of either anti-CD209 blocking antibody or an isotype-matched antibody as negative control. After 3 d, there were fewer CD209^+^ DCs in the blocking antibody group in comparison to the isotype-negative control as measured by flow cytometry (Fig. 9 A). We also evaluated CD25^+^CD4^+^ T cells for the expression of IL-4 and IL-13 by flow cytometry, as we reasoned that peanut-specific T cells would express the activation marker CD25 (Kmieciak et al., 2009; Reddy et al., 2004). In the presence of anti-CD209 Ab, we observed a significant decrease in the percentage of CD25^+^CD4^+^ T cells that express IL-4 and IL-13 (Fig. 9, B and C). These data suggest that CD209^+^ DCs contribute to the allergen-specific Th2 response in established peanut allergy in humans.

In summary, our results suggest that allergen-specific T cells and APCs act reciprocally on each another in established IgE-mediated food allergy. Peanut-specific T cells promote the differentiation of CD209^+^ DCs (Figs. 6, 7, and 8). This was further tested in experiments (Fig. 9, B and C) in which blocking CD209^+^ DCs reduced the Th2 response of the same peanut-specific T cells.

### OIT reduces CD209^+^ DCs in PA patients

OIT was recently approved by the Food and Drug Administration for the treatment of peanut allergy. As we have previously published data that the peanut-specific Th2 population is reduced in peanut OIT (Syed et al., 2014), we then set out to determine whether the CD209^+^ DC population is modulated by OIT. Therefore, we analyzed PBMCs from six PA children before peanut OIT treatment, during OIT up-dosing at 300 mg of peanut protein, and at a maintenance dose of 4,000 mg (Table S11). PBMCs were incubated either with or without peanut protein and then analyzed by flow cytometry. After 3 d, we observed a significant decrease in CD209^+^ DCs at the 300-mg up-dosing phase of OIT in comparison to baseline (Fig. 9, D and E). There was a trend toward a further decrease in CD209^+^ DCs at the maintenance dose of 4,000 mg. We also measured the frequency of peanut-activated CD4^+^ T cells (CD25^+^CD154^+^) and observed a parallel and significant decrease at the 300-mg up-dosing phase of OIT in comparison to baseline (Fig. 9, F and G). Again, there was a trend toward a further decrease in peanut-activated CD4^+^ T cells by the time a maintenance dose of 4,000 mg was reached. These results show that the treatment of peanut allergy with OIT is associated with a decrease in CD209^+^ DCs.

## Discussion

The results presented here provide an integrated picture of the allergen-specific immune response in established IgE-mediated peanut allergy in humans. In allergic, but not NA, individuals, exposure to peanut allergen results in the differentiation of two subsets of DCs: CD209^+^ MDDCs and CD23^+^ myeloid DCs. This APC differentiation is driven by CD4^+^ T cells in an IL4RA-dependent manner. Interestingly, both CD209 and CD23 have been reported to facilitate antigen presentation. In the case of CD209^+^ MDDCs, we show that blocking antibodies against CD209 dampen the Th2 response of allergen-specific CD4^+^ T cells within 3 d of peanut exposure. Thus, the innate and adaptive immune systems act reciprocally on each other to promote the allergic immune response in established food allergy: allergen-specific T cells drive the differentiation of specific APC subclasses, while these APCs act in turn on allergen-specific T cells to boost Th2 cytokine expression. This positive feedback loop may contribute to the persistence of IgE-mediated food allergy. In support of this model, we show clinically that the treatment of peanut allergy with OIT is associated with a decrease in CD209^+^ DCs, consistent with the idea that disrupting this positive feedback loop impairs the allergic immune response.

The work presented here is distinct from findings in the literature. The two studies that introduced a connection between monocytes and food allergy by Zhang et al. (2016) and Neeland et al. (2018) were part of the impetus for our examination of APCs in this study. While Zhang et al. (2016) dealt with risk factors in the development of food allergy, we focus on established food allergy. Unlike both monocyte studies, which used nonspecific LPS for stimulation, we stimulated PBMCs specifically with peanut allergen and show that two specific APC subsets (CD23^+^ myeloid DCs and CD209^+^ MDDCs) are induced only in PBMCs from allergic patients. Our results in humans showing the relevance of IL4RA in this differentiation also complements the work of others in murine models (Tachdjian et al., 2010).

Another important finding presented here is related to the process of antigen presentation (as opposed to cytokine secretion). CD23 is the low-affinity IgE receptor, which, when present on the plasma membrane, can bind and internalize IgE-antigen immune complexes (Hibbert et al., 2005; Sharquie et al., 2013). This IgE receptor is expressed on multiple cell types, including B lymphocytes, monocytes, and DCs, and IL-4 induces CD23 expression via STAT6 (Goenka and Kaplan, 2011). On B cells, CD23 may either facilitate the internalization and subsequent MHCII presentation of allergen to allergen-specific T cells (Selb et al., 2017) or help transfer antigen to DCs for presentation (Engeroff et al., 2018). The induction of CD23 on myeloid DCs after peanut exposure may facilitate the presentation of immune complexes of IgE and peanut allergen in a manner parallel to that seen in B cells, a notion that is supported by a report that the monoclonal anti-CD23 antibody lumiliximab modulates APCs and dampens allergen-induced Th2 immune responses (Poole et al., 2005).

Similar to the low-affinity IgE receptor, CD209 is also involved in antigen presentation. CD209 is a C-type lectin receptor, a class of molecules that aid in the endocytosis of antigen before processing and presentation by APCs (Geijtenbeek et al., 2000). CD209 binds two classes of carbohydrate structures, high-mannose glycans and fucosylated glycans (Menon et al., 2009), and has been shown to bind multiple allergens. These include environmental allergens such as Der p 1 and Der p 2 from house dust mites and Can f 1 from dogs (Emara et al., 2012). Among foods, it has been reported that CD209 preferentially binds proteins derived from allergenic foods, such as peanut or walnut, in comparison to food less commonly associated with allergy, such as pine nuts or chickpeas (Kamalakannan et al., 2016),(Shreffler et al., 2006). In a different experimental system using MDDCs derived from NA individuals after 6-d treatment with IL-4 and GM-CSF, Shreffler et al. (2006) showed that the subsequent introduction of peanut antigen promotes the development of Th2 cells. However, the link was attributed indirectly to CD209 by using a CD209 transfected RAJI cell line to show interaction with Ara h 1.

While understanding the factors leading to the development of food allergy is clearly important, it is also useful to study the immune mechanisms underlying established food allergy and food allergy therapy–induced modifications of the immune system. We show here that allergen exposure leads within days to the development of APCs expressing molecules that promote the capture of allergen bound either by IgE via CD23 or more directly via CD209 by carbohydrate residues. In the case of CD209, we show that a positive feedback loop between allergen-specific T cells and CD209^+^ MDDCs likely occurs that strengthens the Th2 response (Fig. 1). Understanding these factors provides a useful new framework in which to investigate etiologies and treatments for established food allergy.

## Materials and methods

### Study participants

Peanut allergy–discordant twin siblings, PA nontwin pediatric participants, and NA nontwin pediatric participants were recruited at the Sean N. Parker Center for Allergy and Asthma Research at Stanford University. Patient demographics, food allergy history, atopic history, and peanut-specific IgEs are summarized in Table S1, Table S4, Table S5, Table S6, Table S7, Table S8, Table S9, Table S10, and Table S11. Peanut allergy was confirmed by double-blind, placebo-controlled food challenge. Peanut OIT subjects were taken from a convenience and random sampling of participants in a phase 2, randomized controlled study that was previously published (Chinthrajah et al., 2019). All studies were approved by the Institutional Review Board of Stanford University (ClinicalTrials.gov NCT02103270 and NCT01613885). Informed consent was obtained from all patients or their caregivers. Three buffy coats from healthy NA individuals from the Stanford Blood Bank (under approved consent ethical guidelines) were used.

Luminex-based 62-multiplex assay and mass cytometry were used to quantify cytokine secretion and characterize the cellular markers for six pairs of peanut allergy–discordant twins, of whom five pairs were monozygotic and one pair was dizygotic (Table S1). Analyzing twin pairs minimizes the confounding influence of genetic-associated variations between NA and PA participants. To verify that our initial findings in twins were also valid in the general population, we tested PBMCs from 16 PA and 20 NA participants who were age matched (Table S1). To further characterize APCs in response to peanut protein for NA and PA participants, PBMCs from nontwin subjects were cultured in vitro for CFSE labeling, antibody blocking, and T cell depletion experiments (Table S4, Table S5, Table S6, Table S7, Table S8, Table S9, Table S10, and Table S11). The number of NA and PA subjects used for each assay is indicated in the figure legends and was determined by the availability of cryopreserved PBMCs.

### Cell preparation

For the PA subjects shown in Table S1, Table S4, Table S5, Table S6, Table S7, Table S8, Table S9, and Table S10, blood samples were taken from the allergic subjects avoiding peanut at baseline (with the exception of recent oral food challenge). For the peanut subjects shown in Table S11, the blood samples were taken from the allergic subjects before peanut OIT treatment (at baseline), during OIT up-dosing at 300 mg of peanut protein, and at a maintenance dose of 4,000 mg. PBMCs were isolated from blood samples by density gradient centrifugation over Ficoll-Paque, cryopreserved in 10% DMSO in FCS, and stored in liquid nitrogen.

### Cell isolation using magnetic sorting

T cell–depleted PBMCs were obtained by using CD3 magnetic microbeads (Miltenyi Biotec) to remove CD3^+^ T cells from PBMCs. Monocytes and nonmonocytes were enriched from PBMCs using two different methods in parallel. Half of PBMCs were used for untouched monocyte selection using a pan monocytes isolation kit (Miltenyi Biotec), whereas the other half of PBMCs were used for nonmonocyte enrichment using CD14 MicroBeads (Miltenyi Biotec) to remove CD14^+^ cells from PBMCs.

### CFSE staining

The purified untouched monocytes were incubated with CFSE at a final concentration at 1 µM for 5 min at RT. After incubation, staining was terminated by adding warm FBS to a final concentration of 20%, and cells were washed with PBS and resuspended in complete RPMI (RPMI 1640, 5% human serum, and 1% penicillin/streptomycin). These CFSE-labeled monocytes were mixed with nonmonocytes.

### In vitro stimulation

The peanut proteins added to cell culture are derived from the peanut flour used for double-blind, placebo-controlled food challenge in clinic. The peanut flour was dissolved in PBS and sterilized by filtration. The peanut protein concentration was determined by BCA Protein Assay (Pierce). The endotoxin level of peanut protein was assessed by fluorescence-based recombinant factor C assay (Indoor Biotechnologies); the endotoxin level of peanut protein exhibited in cell culture was 0.05 EU/ml.

PBMCs from NA and PA subjects were thawed and plated in each well of 96-well round-bottomed plates with ∼4–5 × 10^5^ cells in 200 µl of complete RPMI per well. The PBMCs were plated in equal numbers per each culture condition for the same sample, and the number of PBMCs plated in the culture was determined by the available quantity of the cryopreserved PBMCs from different samples. After overnight resting of thawed PBMCs, CD3-depleted PBMCs or mixed cells (CFSE-labeled purified monocytes and nonmonocytes) were cultured in the presence or absence of peanut protein for 3 d. Peanut protein was added to the cell culture at a final concentration of 100 µg/ml once on day 1. It is known that a decrease in temperature from 37°C to 4°C can reduce cell-substratum adhesion strength, and cold PBS has been used for collecting the cultured adherent cells (Malheiro et al., 2017). After 3-d culture, the cells were harvested, followed by further washing of the plate with cold PBS three times. In mass cytometry experiments, brefeldin A (1 µg/ml; BioLegend) was included during the last 4 h of the cell culture to inhibit intracellular transport and allow for the detection of cytokines in PBMCs. For the experiment in which antibody to CD209 was added, we wanted to ensure that the expression of Th2 cytokines could be detected in each sample included in this analysis. After 3-d culture, the cells were incubated with PMA (20 ng/ml) and ionomycin (1 µg/ml) in the presence of brefeldin A during the last 4 h of the cell culture. The expression of CD25 (late activation marker) did not change with 4-h PMA and ionomycin stimulation (Reddy et al., 2004). We compared the peanut-activated CD4^+^ T cells producing Th2 cytokines as measured by CD4^+^CD25^+^IL-4^+^ and CD4^+^CD25^+^IL-13^+^ for this experiment. In some cases, anti-IL4RA antibody (clone #25463, 250 µg/ml; R&D Systems), anti-GM-CSF antibody (clone #3209, 250 µg/ml; R&D Systems), mouse IgG1 isotype control (clone #11711, 250 µg/ml; R&D Systems), or mouse IgG2A isotype control (clone #20102, 250 µg/ml; BD Biosciences) was added 3 h before peanut protein stimulation. In some cases, anti-CD209 antibody (clone #120507, 10 µg/ml, R&D Systems) or mouse IgG2B isotype control (clone #20116, 10 µg/ml; R&D Systems) was added in the culture for 3 d (30 min before peanut protein stimulation, 10 µg/ml per day). In some cases, PBMCs were cultured with cow’s milk protein (100 µg/ml), recombinant human IL-4 (500 U/ml; R&D Systems), recombinant human IL-13 (50 U/ml; R&D Systems), recombinant human IL-5 (500 U/ml; R&D Systems), recombinant human GM-CSF (1,000 U/ml; R&D Systems), or human IL-4 combined with human GM-CSF for 3 d.

### Cytokine assays

Cryopreserved PBMCs from six pairs of twins discordant for peanut allergy were thawed. For each condition of this experiment, PBMCs were cultured at 5 × 10^5^ cells per 200 µl. After 3-d culture, the supernatants were harvested and stored at −80°C. The secretion level of cytokines or chemokines from PBMCs in supernatants were measured using 62-multiplex assay on the Luminex 200 IS system (Affymetrix) by the Stanford Human Immune Monitoring Center. All samples were tested in duplicate wells. Data were analyzed using MasterPlex software (Hitachi Software Engineering America; MiraiBio Group), and the average of two median fluorescence intensity (MFI) values for each sample for each analyte were reported by the Stanford Human Immune Monitoring Center. The ratios were calculated by dividing the average MFI of each analyte for each sample by the average MFI of each analyte for complete RPMI medium control. These ratios were used to present the secretion level of each cytokine or chemokine from PBMCs for each sample.

### Flow cytometry

Cells were collected after 3-d culture. Before surface staining, Fc receptors were blocked with Human TruStain FcX (BioLegend). Cells (∼1–2 × 10^6^) were stained in 100 µl volume with cell surface antibodies and viability dyes for 30 min on ice, followed by washes in FACS buffer (PBS with 0.25% BSA and 1 mM EDTA). The viability dyes propidium iodide (BioLegend) and Aqua (Thermo Fisher Scientific) were used for cell surface staining and intracellular staining, respectively. For staining of cytoplasmic cytokines or antigens, cells were fixed with 2% paraformaldehyde overnight at 4°C, and then washed twice with permeabilization buffer (eBiosciences). Permeabilized cells were stained with intracellular antibodies for 30 min at RT followed by washing once with permeabilization buffer and once with FACS buffer. All samples were run on a Cytek flow cytometer and analyzed using FlowJo v10.6.0 software. The antibodies used for flow cytometry staining are listed in Table S12.

### Mass cytometry (antibody preparation, sample preparation, data acquisition, and data analysis)

Antibodies purchased from Fluidigm or conjugated with the lanthanides using MAXPAR antibody labeling kit (Fluidigm) following the manufacturer’s protocol are listed in Table S3. Antibodies were titrated before use.

Cells were collected after 3-d culture and stained for viability with 5 µM cisplatin in PBS (Fluidigm) for 5 min at RT. The cisplatin staining was quenched with Maxpar Cell Staining Buffer (Fluidigm). Before surface staining, Fc receptors were blocked with Human TruStain FcX (BioLegend), and cells were stained with cell surface antibodies for 45 min on ice. Following cell surface staining, cells were washed with Maxpar Cell Staining Buffer twice and fixed in 2% paraformaldehyde overnight at 4°C. The cells were washed twice in permeabilization buffer (eBiosciences) and then stained with intracellular antibodies at RT for 45 min. Cells were washed twice with permeabilization buffer and once with Maxpar Cell Staining Buffer and then incubated in 0.0625 µM iridium intercalator (Fluidigm) for 20 min at RT. Cells were washed twice with Maxpar Cell Staining Buffer and twice with PBS and then stored in PBS at 4°C until acquisition.

Before acquisition, cells were washed twice with deionized water. Cells were resuspended at a concentration of 0.5 million cells/ml in deionized water containing a one-quarter dilution of EQ 4 Element Beads (Fluidigm), followed by filtering through a 35-µm strainer to remove aggregates. The samples were acquired at an event rate of <500 events/s on a Helios (Fluidigm) at the Sean N. Parker Center for Allergy and Asthma Research at Stanford University. After acquisition, the data were normalized using bead-based normalization in CyTOF software. The intact live cells were gated to exclude normalization beads, dead cells, and doublets using FlowJo v10.6.0. For all analyses on FCS files from mass cytometry, marker expression values were transformed using the inverse hyperbolic sine (arcsinh) transformation function from the Cytofkit R package (Chen et al., 2016).

Unsupervised computational analysis was performed for the PBMC samples cultured with medium alone or with peanut protein from peanut allergy–discordant twin siblings. In each sample, 50,000 cells of the pregated live, single cells were randomly selected, and the marker expression values were arcsinh transformed with a cofactor of 5. Unsupervised clustering was performed on the expression values of the markers (CD3, CD4, CD8, CD45RA, CCR7, CD27, CXCR3, CCR4, CCR6, CD19, CD20, CD56, CD14, CD16, HLA-DR, CD11c, CD123, and CD57) using the FlowSOM algorithm (Crowell et al., 2020; R package Catalyst, v1.14.0), which uses a self-organizing map followed by hierarchical consensus meta-clustering to detect cell populations (Van Gassen et al., 2015). Default parameters and a predetermined number of 40 clusters were used. The median levels of the lineage markers across all cells per cluster were visualized in a heatmap (R package ComplexHeatmap, v2.6.2). The main subtypes of immune cells were identified based on the median expression levels of markers in each cluster. We also applied the nonlinear dimensionality reduction technique, UMAP, to the expression levels of markers from a set of 12,000 randomly selected cells (500 cells per file, 24 files) using the R package UMAP (R package Catalyst, v1.14.0; default parameters) for visualization of the high-dimensional data.

### Statistical analysis

We compared different treatments in vitro from the same participant using Wilcoxon signed rank comparisons test (nonparametric paired test, two sided) and the same treatments in vitro in PA participants versus NA participants using Wilcoxon rank sum comparisons test (nonparametric unpaired test, two sided). The percentage change in cell frequency or protein expression intensity was calculated as the difference between the average values with or without peanut stimulation. For the Luminex cytokine analysis, P values were adjusted for multiple comparisons using the Benjamini and Hochberg approach to control the FDR (Benjamini and Hochberg, 1995), and FDR-adjusted P < 0.1 was considered significant.

Statistical analyses were performed using Wilcoxon test function in R software. PCA function from FactoMineR and factoextra R package and distance function in R software were employed for PCA and Euclidean distances analysis, respectively. PERMANOVA was applied to examine the contribution of variables to the separation of the data in multiple dimensional space using adonis2 function in the vegan package in R. Heatmaps were generated using ComplexHeatmap R package (Gu et al., 2016). All dot plots overlaid with boxplots and line connections were compiled with ggplot2 R package or Prism 8 (GraphPad).

### Online supplemental material

Table S1, Table S4, Table S5, Table S6, Table S7, Table S8, Table S9, Table S10, and Table S11 are demographic tables that characterize the subjects who donated PBMCs for experiments. Table S2 presents the adjusted and unadjusted P values in the comparison analysis of 62 cytokines measured by Luminex assay for PBMCs from allergy-discordant twins. Table S3 depicts the staining panels for CyTOF. Table S12 depicts the antibodies and reagents for flow cytometry experiments. Fig. S1 shows no change in total live cell numbers when PBMCs are incubated with or without peanut protein for 3 d or when the subjects are PA or NA subjects and the immune cell types identified using unsupervised FlowSOM-based clustering analysis. Fig. S2 shows the mass cytometric gating strategies for monocytes and CD11c^+^ DCs; the expression of CD86 and HLA-DR on monocytes versus Lin^−^CD14^−^CD16^−^HLA^−^DR^+^ cells; and the percentage of CD11c^+^ DCs per total PBMCs for both NA and PA participants. Fig. S3 shows the CFSE-based experiments for tracking monocytes after peanut protein exposure for PA subjects. Fig. S4 lays out the mass cytometry plots, which show that CD23^+^CD11c^+^ DCs are induced by peanut protein in PA but not NA participants. Fig. S5 shows that Th2 cytokines promote monocyte differentiation into CD209^+^ DCs, and blocking GM-CSF partially reverses the decrease in monocyte frequency induced by peanut protein in PA participants.

## Supplementary Material

Table S1 contains the demographics for twin siblings and nontwin individuals analyzed in Figs. 2, 3, 7, S1, and S2.

Table S2 is a comparison of 62 cytokines secreted by PBMCs cultured with or without peanut protein from peanut allergy–discordant twins.

Table S3 shows staining panels for mass cytometry.

Table S4 contains demographic information for nontwin individuals analyzed in Fig. 3, G and H.

Table S5 lists demographic information for nontwin individuals analyzed in Fig. 4, A–C; and Fig. 8.

Table S6 contains the demographics for nontwin individuals analyzed in Fig. 4, D–F; Fig. 6, A and B; and Fig. S3.

Table S7 lists demographic information for nontwin individuals analyzed in Fig. 5, A, B, and E.

Table S8 includes the demographics for nontwin individuals analyzed in Fig. 5, C and D; Fig. 6, D–G; Fig. S4; and Fig. S5 H.

Table S9 lists demographic information for nontwin individuals analyzed in Fig. 5, F and G.

Table S10 shows the demographics for nontwin individuals analyzed in Fig. 9, A, B, and C.

Table S11 contains demographic information for nontwin individuals analyzed in Fig. 9, D–G.

Table S12 lists antibodies and reagents used for flow cytometry experiments.
