# Supplementary material for: A positive feedback loop reinforces the allergic immune response in human peanut allergy
Source: J Exp Med. 2021 May 4;218(7):e20201793. doi: 10.1084/jem.20201793 (PMC8103542; doi:10.1084/jem.20201793)
Supplement: Table S1 — contains the demographics for twin siblings and nontwin individuals analyzed in Figs. 2, 3, 7, S1, and S2. [file JEM_20201793_TableS1.docx]

**Table S1.**Demographics for twin siblings and non-twin individuals analyzed in Fig. 2, Fig. 3, Fig. 7, Fig. S1, and Fig.S2 (Luminex assay and mass cytometry experiment)
