# Supplementary material for: A positive feedback loop reinforces the allergic immune response in human peanut allergy
Source: J Exp Med. 2021 May 4;218(7):e20201793. doi: 10.1084/jem.20201793 (PMC8103542; doi:10.1084/jem.20201793)
Supplement: Table S3 — shows staining panels for mass cytometry. [file JEM_20201793_TableS3.docx]

**Table S3.** Staining panels for mass cytometry
