# Supplementary material for: A positive feedback loop reinforces the allergic immune response in human peanut allergy
Source: J Exp Med. 2021 May 4;218(7):e20201793. doi: 10.1084/jem.20201793 (PMC8103542; doi:10.1084/jem.20201793)
Supplement: Table S5 — lists demographic information for nontwin individuals analyzed in Fig. 4, A–C; and Fig. 8. [file JEM_20201793_TableS5.docx]

**Table S5.**Demographics for non-twin individuals analyzed in Fig. 4, A–C and Fig. 8. (evaluation of CD209^+^CD11c^+^ DCs in total PBMCs or CD3-depleted PBMCs by flow cytometry)
