# Supplementary material for: A positive feedback loop reinforces the allergic immune response in human peanut allergy
Source: J Exp Med. 2021 May 4;218(7):e20201793. doi: 10.1084/jem.20201793 (PMC8103542; doi:10.1084/jem.20201793)
Supplement: Table S6 — contains the demographics for nontwin individuals analyzed in Fig. 4, D–F; Fig. 6, A and B; and Fig. S3. [file JEM_20201793_TableS6.docx]

**Table S6.**Demographics for non-twin individuals analyzed in Fig. 4, D–F; Fig. 6, A and B; and Fig. S3 (evaluation of CD209^+^CD11c^+^ DCs for the experiments including CFSE labeling and antibody blocking by flow cytometry)
