# Supplementary material for: A positive feedback loop reinforces the allergic immune response in human peanut allergy
Source: J Exp Med. 2021 May 4;218(7):e20201793. doi: 10.1084/jem.20201793 (PMC8103542; doi:10.1084/jem.20201793)
Supplement: Table S8 — includes the demographics for nontwin individuals analyzed in Fig. 5, C and D; Fig. 6, D–G; Fig. S4; and Fig. S5 H. [file JEM_20201793_TableS8.docx]

**Table S8.**Demographics for non-twin individuals analyzed in Fig. 5, C and D; Fig. 6, D–G; Fig. S4; and Fig. S5 H (evaluation of CD23^+^CD11c^+^ DCs for antibody blocking experiments by mass cytometry)
