# Supplementary material for: A positive feedback loop reinforces the allergic immune response in human peanut allergy
Source: J Exp Med. 2021 May 4;218(7):e20201793. doi: 10.1084/jem.20201793 (PMC8103542; doi:10.1084/jem.20201793)
Supplement: Table S9 — lists demographic information for nontwin individuals analyzed in Fig. 5, F and G. [file JEM_20201793_TableS9.docx]

**Table S9.**Demographics for non-twin individuals analyzed in Figure 5, F and G (evaluation of CD23^+^CD11c^+^ DCs for CFSE labeling experiments by flow cytometry)
