# Supplementary material for: A positive feedback loop reinforces the allergic immune response in human peanut allergy
Source: J Exp Med. 2021 May 4;218(7):e20201793. doi: 10.1084/jem.20201793 (PMC8103542; doi:10.1084/jem.20201793)
Supplement: Table S10 — shows the demographics for nontwin individuals analyzed in Fig. 9, A, B, and C. [file JEM_20201793_TableS10.docx]

**Table S10.**Demographics for non-twin individuals analyzed in Fig. 9, A–C (evaluation of CD209^+^CD11c^+^ DCs for antibody blocking experiments by flow cytometry)
